# Global, regional, and national burden of headache disorders, 1990–2021, with forecasts to 2050: A Global Burden of Disease study 2021

**DOI:** 10.1016/j.xcrm.2025.102348

**Published:** 2025-09-18

**Authors:** GBD 2021 Headache Collaborators, Tissa Wijeratne, Jiyeon Oh, Soeun Kim, Yesol Yim, Min Seo Kim, Jae Il Shin, Yun-Seo Oh, Raon Jung, Yun Seo Kim, Lee Smith, Hasan Aalruz, Rami Abd-Rabu, Deldar Morad Abdulah, Richard Gyan Aboagye, Meysam Abolmaali, Dariush Abtahi, Ahmed Abualhasan, Rufus Adesoji Adedoyin, Qorinah Estiningtyas Sakilah Adnani, Fatemeh Afrashteh, Navidha Aggarwal, Danish Ahmad, Ali Ahmadi, Negar Sadat Ahmadi, Amir Mahmoud Ahmadzade, Syed Anees Ahmed, Salah Al Awaidy, Sawsan Alabbad, Muaaz M. Alajlani, Yazan Al-Ajlouni, Mohammed Usman Ali, Syed Shujait Ali, Waad Ali, Joseph Uy Almazan, Najim Z. Alshahrani, Awais Altaf, Mohammad Al-Wardat, Karem H. Alzoubi, Sohrab Amiri, Hubert Amu, Ganiyu Adeniyi Amusa, David B. Anderson, Saleha Anwar, Demelash Areda, Mohammad Asghari-Jafarabadi, Sait Ashina, Javed Ashraf, Tahira Ashraf, Ali Azargoonjahromi, Yogesh Bahurupi, Atif Amin Baig, Soham Bandyopadhyay, Mainak Bardhan, Hiba Jawdat Barqawi, Azadeh Bashiri, Mohammad-Mahdi Bastan, Maryam Bemanalizadeh, Isabela M. Bensenor, Alemshet Yirga Yirga Berhie, Akshaya Srikanth Bhagavathula, Sonu Bhaskar, Vivek Bhat, Gurjit Kaur Bhatti, Jasvinder Singh Bhatti, Cem Bilgin, Atanu Biswas, Bruno Bizzozero-Peroni, Yasser Bustanji, Luis Alberto Cámera, Edoardo Caronna, Andre F. Carvalho, Sandip Chakraborty, Patrick R. Ching, Nikos Christodoulou, Dinh-Toi Chu, Hongyuan Chu, Natalia Cruz-Martins, Omid Dadras, Xiaochen Dai, Emanuele D'Amico, Amira Hamed Darwish, Sindhura Deekonda, Vinoth Gnana Chellaiyan Devanbu, Samath Dhamminda Dharmaratne, Adriana Dima, Temesgien Ergetie Dinkayehu, Huyen Do, Paul Narh Doku, Ojas Prakashbhai Doshi, Abdel Rahman E’mar, Negin Eissazade, Chadi Eltaha, Ayesha Fahim, Jawad Fares, Mohsen Farjoud Kouhanjani, Andre Faro, Patrick Fazeli, Seyed-Mohammad Fereshtehnejad, Pietro Ferrara, Nuno Ferreira, Florian Fischer, Arianna Fornari, Márió Gajdács, Miglas Welay Gebregergis, Delaram J. Ghadimi, Amir Ghaffari Jolfayi, Elena V. Gnedovskaya, Mahaveer Golechha, Enrique Gomez Figueroa, Mohammad Hashem Hashempur, Md Saquib Hasnain, Amr Hassan, Nageeb Hassan, Mahgol Sadat Hassan Zadeh Tabatabaei, Mohamed I. Hegazy, Golnaz Heidari, Bartosz Helfer, Md Mahbub Hossain, Mowafa Househ, Chengxi Hu, Ivo Iavicoli, Olayinka Stephen Ilesanmi, Irena M. Ilic, Muhana Fawwazy Ilyas, Salim Ilyasu, Nahlah Elkudssiah Ismail, Ali Jafari-Khounigh, Haitham Jahrami, Manthan Dilipkumar Janodia, Ruwan Duminda Jayasinghe, Bijay Mukesh Jeswani, Jost B. Jonas, Nitin Joseph, Rizwan Kalani, Moien A.B. Khan, Sorour Khateri, Mahalaqua Nazli Khatib, Hamid Reza Khayat Kashani, Feriha Fatima Khidri, Moein Khormali, Sepehr Khosravi, Yun Jin Kim, Farzad Kompani, Karel Kostev, Kewal Krishan, Bindu Krishnan, Barthelemy Kuate Defo, Mohammed Kuddus, Mukhtar Kulimbet, Rakesh Kumar, Vijay Kumar, Ville Kytö, Savita Lasrado, Seung Won Lee, Jacopo Lenzi, Matilde Leonardi, Giancarlo Lucchetti, Alessandra Lugo, Irsa Fatima Makhdoom, Arashk Mallahzadeh, Vahid Mansouri, Roy Rillera Marzo, Yasith Mathangasinghe, Mahsa Mayeli, Asim Mehmood, Atte Meretoja, Tomislav Mestrovic, Sachith Mettananda, Giuseppe Minervini, Archana Mishra, Prasanna Mithra, Khabab Abbasher Hussien Mohamed Ahmed, Ibrahim Mohammadzadeh, Shafiu Mohammed, Lorenzo Monasta, Shane Douglas Morrison, Amin Nabavi, Zuhair S. Natto, Javaid Nauman, Luciano Nieddu, Fred Nugen, Andrew T. Olagunju, Arão Belitardo Oliveira, Welber Sousa Oliveira, Hany A. Omar, Goran Latif Omer, Nikita Otstavnov, Mahesh P A, Leonidas D. Panos, Romil R. Parikh, Shankargouda Patil, Apurba Patra, Paolo Pedersini, Umberto Pensato, Prince Peprah, Mario F.P. Peres, Michael A. Piradov, Patricia Pozo-Rosich, Jalandhar Pradhan, Sanjay Prakash, Akila Prashant, Jagadeesh Puvvula, Alireza Rafiei, Alberto Raggi, Amir Masoud Rahmani, Mahmoud Mohammed Ramadan, Devarajan Rathish, Ilari Rautalin, Salman Rawaf, Mohsen Rezaeian, Donya Rezazadeh Eidgahi, Taeho Gregory Rhee, Priyanka Roy, Adnan Saad Eddin, Cameron John Sabet, Basema Ahmad Saddik, Erfan Sadeghi, Umar Saeed, Fatemeh Saheb Sharif-Askari, Narjes Saheb Sharif-Askari, Pragyan Monalisa Sahoo, Sohrab Salimi, Abdallah M. Samy, Jennifer Saulam, Monika Sawhney, Yigit Can Senol, Subramanian Senthilkumaran, Yashendra Sethi, Homa Seyedmirzaei, Mahan Shafie, Anas Shamsi, Amin Sharifan, Hatem Samir Shehata, Rekha Raghuveer Shenoy, Farhad Shokraneh, Jaspreet Kaur Sidhu, Baljinder Singh, Harmanjit Singh, Jasvinder A. Singh, Surjit Singh, Anna Aleksandrovna Skryabina, Farrukh Sobia, Bahadar S. Srichawla, Vinay Suresh, Chandan Kumar Swain, Sree Sudha T Y, Payam Tabaee Damavandi, Celine Tabche, Mohammad Tabish, Manoj Tanwar, Mohamad-Hani Temsah, Masayuki Teramoto, Nghia Minh Tran, Thang Huu Tran, Aristidis Tsatsakis, Aniefiok John Udoakang, Jibrin Sammani Usman, Hande Uzunçıbuk, Jef Van den Eynde, Tommi Juhani Vasankari, Narayanaswamy Venketasubramanian, Jorge Hugo Villafañe, Lintao Wang, Xingxin Wang, Yuan-Pang Wang, Taweewat Wiangkham, Andrea Sylvia Winkler, Alemayehu Molla Wollie, Zheman Xiao, Yazachew Engida Engida Yismaw, Abdilahi Yousuf, Zhongyi Zhao, Magdalena Zielińska, Min Kyung Chu, Tae-Jin Song, Dong Keon Yon, Valery L. Feigin

**Keywords:** Global Burden of Disease, headache disorders, migraine, tension-type headache, disability-adjusted life years

## Abstract

Headache disorders, especially migraines and tension-type headaches (TTHs), are major global public health concerns, as shown by the Global Burden of Diseases, Injuries, and Risk Factors Study (GBD) 2021. We provide updated global estimates of prevalence and years lived with disability (YLDs) from 1990 to 2021 across 204 countries and territories and forecasts through 2050. In 2021, there are 2.0 billion people with TTH and 1.2 billion with migraine. Although TTH is more prevalent, migraine causes higher disability. While crude prevalence and YLDs increased, age-standardized rates remained stable and are projected to continue this trend due to population growth. There is a disproportionately higher burden in women aged 30–44 and countries with higher Socio-demographic Index and Healthcare Access and Quality Index. Despite this, migraines remain underrecognized in health policies and funding. This study emphasizes the urgent need to prioritize headache disorders in global health agendas.

## Highlights

• Tension-type headache is more common than migraine in 2021
• Migraine contributes to a more disabling burden than tension-type headaches in 2021
• Headache burden is projected to remain age standardized and stable through 2050
• Females aged 30–44 years experience a disproportionately higher burden in 2021

## Graphical abstract

**Figure:**
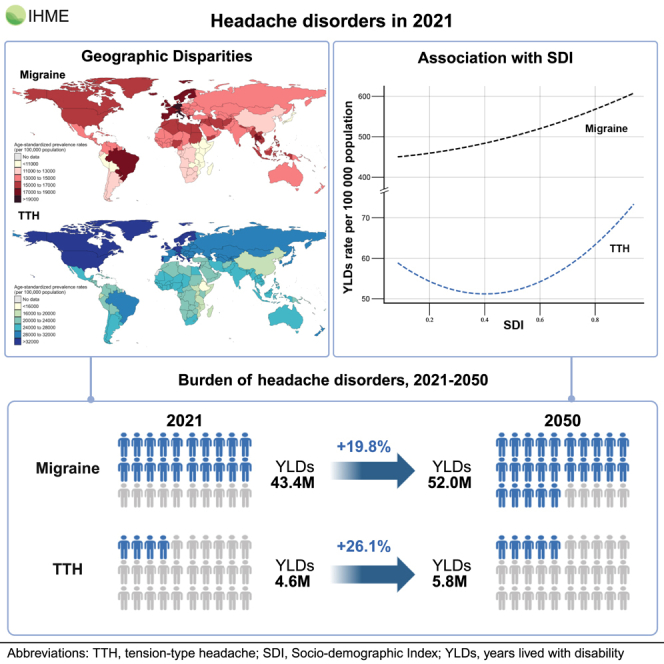

## Teaser

Using GBD 2021, Wijeratne et al. present updated global estimates and 2050 forecasts for migraine and tension-type headaches. Despite stable age-standardized rates, the burden remains high, especially among women aged 30–44 years, underscoring the need to prioritize headache disorders in global health policy and resource allocation.

## Introduction

Migraines and tension-type headaches (TTHs) are prevalent and disabling neurological disorders that contribute significantly to the global headache burden.^1^ Migraine, a recurrent condition characterized by moderate to severe headaches, is often accompanied by reversible neurological and systemic conditions, such as photophobia, phonophobia, skin sensitivity, and gastrointestinal disturbances.^2^^,^^3^ In contrast, TTH is characterized by mild to moderate pain, often described as a tight band around the head,^4^ and presents as a bilateral headache without symptoms such as nausea or vomiting typically seen in migraines.^5^

According to the Global Burden of Diseases, Injuries, and Risk Factors Study (GBD) 2021, headache disorders ranked as the second most prevalent condition worldwide, following oral disorders, and the third leading cause of years lived with disability (YLDs), following low back pain, major depressive disorder, and age-related and other hearing loss.^6^ Migraines and TTH are the most prevalent among headache disorders, affecting diverse populations globally. However, they are often underdiagnosed and undertreated, imposing a significant burden due to their chronic nature and frequent comorbidities, such as sleep disorders, anxiety, and depression, underscoring their critical role in global health challenges. The COVID-19 pandemic disrupted efforts to reduce the burden of headache disorders, with many individuals experiencing increased triggers, such as stress, reduced access to healthcare, and lifestyle changes.^7^ Evidence suggests that SARS-CoV-2 infection caused acute or chronic headaches, while vaccination occasionally triggered transient migraine-like episodes.^8^^,^^9^^,^^10^ However, the duration and generalizability of the impact of the pandemic on headache disorders remain unclear and may take time to fully emerge.

The significance of this study lies in its comprehensive analysis of all currently available GBD data, offering estimates of the disease burden from 1990 to 2021 across different age groups, sexes, geographical regions, Socio-demographic Index (SDI), and the Healthcare Access and Quality (HAQ) Index. In addition, to provide valuable insights into future trends, we also projected YLD counts and rates up to 2050. Understanding the burden estimates can allow healthcare providers, policymakers, and researchers to allocate resources better, prioritize interventions, and tailor treatment strategies to reduce the burden of headache disorders. This manuscript was produced as part of the GBD Collaborator Network and following the GBD Protocol.^11^

## Results

### Overview

In 2021, headache disorders were the second most prevalent disease globally, following oral disorders, affecting an estimated 2.8 billion (95% uncertainty interval [UI], 2.6–3.0) individuals. TTH was the second most common specific condition (2.0 billion [1.8–2.3]), while migraine was the seventh (1.2 billion [95% UI, 1.0–1.3]). Headache disorders were also the third leading cause of YLDs among all conditions in GBD 2021 (48.0 million [95% UI, 9.8–101.0]), following low back pain and depressive disorders. In terms of incidence, there were 809.2 million (95% UI, 717.8–896.0) incident cases in 2021, with an age-standardized rate of 10,084.5 (8,956.5–11,170.8) per 100,000.

### Global, regional, and national burden of migraine, 1990–2021

In 2021, the global prevalence of migraine was estimated at 1.2 billion cases (95% UI, 1.0–1.3), reflecting a 58.1% increase from 732.6 million cases (624.6–847.1) in 1990. While the absolute number of migraine cases has grown significantly, the global age-standardized prevalence rate of migraines experienced a marginal 1.6% (95% UI, 0.3–2.6) increase, from 14,027.7 per 100,000 in 1990 to 14,246.6 per 100,000 in 2021 (Table 1). Similarly, the number of YLDs in 2021 reached 43.4 million (95% UI, 6.7–95.1), representing a 58.2% increase from 1990 (27.4 million [4.1–60.3]). The age-standardized YLD rate for migraines remained stable (526.8 per 100,000 in 1990; 532.7 per 100,000 in 2021; Table 1).

**Table 1:** Age-standardized YLD rates of migraine and TTH in 1990, 2021, and 2050 by SDI level, by GBD region, and globally (95% UI)

Location	Prevalence		YLDs		
	2021	Percentage change, 1990–2021	2021	Percentage change, 1990–2021	2050
**Migraine**					

Global	14,246.55 (12,194.12 to 16,378.70)	0.02 (0.00–0.03)	532.70 (80.57–1,167.71)	0.01 (−0.04 to 0.03)	531.80 (81.05–1,165.38)
SDI levels					
High SDI	15,365.14 (13,250.29 to 17,765.44)	0.01 (−0.01 to 0.02)	573.62 (89.78–1,236.04)	0.00 (−0.02 to 0.02)	601.57 (100.89–1,290.92)
High-middle SDI	13,502.73 (11,610.06 to 15,484.75)	0.02 (0.00–0.03)	517.57 (92.95–1,103.35)	0.01 (−0.08 to 0.04)	512.63 (85.46–1,098.01)
Middle SDI	14,344.23 (12,233.37 to 16,477.29)	0.06 (0.04–0.07)	535.85 (74.81–1,178.27)	0.05 (0.02–0.07)	546.97 (77.11–1,203.26)
Low-middle SDI	14,786.99 (12,677.14 to 16,979.01)	−0.00 (−0.02 to 0.01)	544.25 (73.88–1,201.85)	0.00 (−0.02 to 0.02)	547.01 (76.46–1,206.14)
Low SDI	12,808.97 (10,909.26 to 14,754.40)	−0.00 (−0.01 to 0.01)	475.21 (76.39–1,031.51)	0.01 (−0.01 to 0.02)	457.06 (79.27–985.23)
GBD region					
Southeast Asia, East Asia, and Oceania	13,313.82 (11,492.53 to 15,284.36)	0.07 (0.05–0.09)	500.77 (67.92–1,104.95)	0.07 (0.00–0.10)	517.50 (68.89–1,140.37)
East Asia	11,798.36 (10,162.49 to 13,566.96)	0.07 (0.03–0.11)	444.29 (66.76–971.75)	0.07 (−0.00 to 0.11)	444.77 (67.45–973.60)
Oceania	14,043.19 (11,934.61 to 16,427.13)	0.00 (0.00–0.00)	519.02 (64.65–1,151.04)	0.00 (−0.02 to 0.03)	524.35 (65.47–1,163.51)
South Asia	14,859.95 (12,704.02 to 16,953.54)	0.00 (−0.03 to 0.03)	537.87 (68.02–1,198.33)	0.01 (−0.03 to 0.04)	542.09 (68.05–1,207.38)
Central Europe, Eastern Europe, and Central Asia	13,923.11 (11,992.78 to 16,000.50)	−0.01 (−0.01 to −0.00)	547.23 (133.10–1,174.44)	−0.01 (−0.03 to −0.00)	544.11 (128.43–1,172.78)
Central Asia	13,584.23 (11,538.76 to 15,918.11)	−0.01 (−0.01 to −0.00)	507.35 (90.34–1,144.53)	−0.01 (−0.02 to 0.01)	507.14 (90.23–1,145.20)
Central Europe	13,822.04 (11,854.14 to 15,865.31)	−0.00 (−0.01 to −0.00)	525.24 (109.75–1,158.95)	−0.00 (−0.01 to 0.01)	527.05 (109.96–1,163.21)
Eastern Europe	14,077.09 (12,210.58 to 16,112.04)	−0.00 (−0.00 to −0.00)	574.43 (164.13–1,193.90)	−0.00 (−0.01 to 0.01)	574.95 (164.91–1,196.49)
High-income	15,927.19 (13,797.87 to 18,423.50)	0.00 (−0.01 to 0.02)	592.45 (90.70–1,280.41)	−0.00 (−0.02 to 0.02)	597.87 (90.85–1,291.37)
Australasia	13,433.71 (11,438.78 to 15,621.49)	0.00 (0.00–0.00)	498.64 (86.96–1,100.43)	0.00 (−0.02 to 0.03)	499.53 (87.14–1,102.99)
High-income Asia Pacific	11,072.69 (9,484.03 to 12,800.27)	0.01 (−0.03 to 0.04)	422.48 (85.63–937.71)	0.01 (−0.03 to 0.04)	422.07 (85.95–936.20)
High-income North America	16,709.11 (14,462.52 to 19,276.29)	−0.02 (−0.06 to 0.02)	614.00 (89.37–1,352.42)	−0.03 (−0.07 to 0.01)	613.28 (89.39–1,350.77)
Southern Latin America	11,706.98 (9,960.37 to 13,667.23)	0.03 (−0.01 to 0.08)	442.43 (85.81–974.19)	0.02 (−0.03 to 0.07)	442.72 (85.95–974.29)
Western Europe	18,170.70 (15,724.92 to 21,091.55)	0.01 (−0.01 to 0.02)	676.64 (96.68–1,471.91)	0.01 (−0.01 to 0.03)	675.63 (96.78–1,471.35)
Latin America and Caribbean	15,409.21 (13,263.10 to 17,822.77)	0.01 (−0.01 to 0.03)	570.79 (74.56–1,251.75)	0.01 (−0.01 to 0.03)	571.43 (74.72–1,254.99)
Andean Latin America	10,614.71 (8,993.46 to 12,309.15)	0.06 (0.02–0.13)	401.89 (72.75–893.96)	0.06 (−0.01 to 0.12)	405.56 (72.93–903.75)
Caribbean	14,237.12 (12,004.33 to 16,529.90)	−0.00 (−0.00 to −0.00)	528.99 (72.30–1,148.15)	−0.01 (−0.02 to 0.01)	530.77 (72.61–1,152.78)
Central Latin America	14,178.67 (12,097.70 to 16,441.61)	0.01 (−0.01 to 0.04)	531.67 (76.53–1,159.87)	0.01 (−0.02 to 0.04)	531.60 (76.81–1,161.34)
Tropical Latin America	18,595.34 (16,084.24 to 21,444.51)	0.02 (−0.01 to 0.06)	679.39 (73.91–1,542.87)	0.02 (−0.01 to 0.07)	680.10 (74.00–1,544.60)
Sub-Saharan Africa	12,244.68 (10,486.07 to 14,159.33)	0.01 (0.00–0.02)	459.32 (81.66–994.71)	0.01 (−0.00 to 0.02)	464.92 (82.16–1,007.45)
Central Sub-Saharan Africa	12,396.15 (10,491.24 to 14,511.21)	−0.00 (−0.00 to −0.00)	461.37 (80.99–1,008.48)	0.01 (−0.02 to 0.03)	466.43 (81.92–1,017.87)
Eastern Sub-Saharan Africa	9,084.26 (7,732.97 to 10,507.26)	0.01 (−0.00 to 0.02)	347.19 (77.86–757.55)	0.01 (−0.02 to 0.03)	349.98 (78.59–763.53)
Southern Sub-Saharan Africa	12,876.73 (11,102.68 to 14,843.51)	−0.00 (−0.00 to −0.00)	477.47 (85.49–1,025.39)	−0.01 (−0.03 to −0.00)	478.67 (85.39–1,025.96)
Western Sub-Saharan Africa	14,870.35 (12,715.58 to 17,284.92)	0.00 (−0.02 to 0.02)	554.49 (84.75–1,214.04)	0.01 (−0.01 to 0.03)	558.74 (85.54–1,222.08)
North Africa and Middle East	15,304.13 (13,071.93 to 17,749.48)	0.00 (−0.01 to 0.02)	598.94 (108.94–1,305.73)	0.00 (−0.02 to 0.02)	605.59 (109.46–1,318.88)
Southeast Asia	16,180.66 (13,890.15 to 18,730.39)	−0.02 (−0.03 to −0.01)	607.13 (69.70–1,362.67)	−0.02 (−0.03 to 0.01)	609.13 (70.45–1,365.61)

**TTH**					

Global	24,764.77 (21,863.62 to 27,954.74)	−0.01 (−0.01 to 0.00)	55.69 (16.13–185.07)	−0.02 (−0.05 to 0.01)	55.36 (16.03–183.65)
SDI levels					
High SDI	30,603.28 (27,152.87 to 34,513.40)	−0.02 (−0.03 to −0.01)	66.79 (18.15–235.79)	−0.02 (−0.07 to 0.01)	71.94 (20.35–247.94)
High-middle SDI	23,862.61 (21,031.88 to 26,904.35)	−0.01 (−0.02 to 0.01)	59.93 (18.33–179.03)	−0.05 (−0.10 to −0.01)	57.20 (16.57–179.34)
Middle SDI	23,378.72 (20,608.16 to 26,365.06)	0.06 (0.05–0.07)	51.54 (14.95–174.76)	0.05 (−0.02 to 0.14)	52.44 (15.20–177.11)
Low-middle SDI	25,022.79 (22,033.70 to 28,284.15)	0.00 (−0.00 to 0.00)	52.99 (14.84–182.64)	0.01 (−0.06 to 0.07)	53.78 (15.23–183.84)
Low SDI	22,780.22 (19,950.88 to 25,943.94)	−0.01 (−0.01 to −0.01)	51.76 (15.45–171.97)	0.00 (−0.05 to 0.05)	51.48 (15.72–167.07)
GBD region					
Southeast Asia, East Asia, and Oceania	20,715.30 (18,205.92 to 23,454.07)	0.08 (0.05–0.10)	45.87 (13.30–153.94)	0.04 (−0.02 to 0.17)	46.64 (13.40–157.30)
East Asia	18,489.51 (16,320.68 to 20,929.50)	0.08 (0.04–0.12)	43.40 (13.06–141.04)	0.04 (−0.03 to 0.19)	43.41 (13.06–141.25)
Oceania	22,142.50 (18,985.45 to 25,544.46)	0.00 (−0.00 to 0.00)	46.21 (13.06–163.83)	0.00 (−0.09 to 0.09)	46.26 (13.14–164.55)
South Asia	25,405.47 (22,430.09 to 28,583.39)	0.00 (−0.00 to 0.00)	50.98 (13.59–180.49)	0.00 (−0.06 to 0.07)	50.81 (13.52–180.51)
Central Europe, Eastern Europe, and Central Asia	30,801.99 (27,185.92 to 34,791.81)	−0.00 (−0.01 to 0.01)	84.31 (26.91–262.98)	−0.02 (−0.05 to 0.01)	82.59 (25.96–259.86)
Central Asia	30,274.55 (26,183.72 to 34,528.86)	−0.00 (−0.00 to 0.00)	66.89 (18.09–236.36)	−0.00 (−0.05 to 0.04)	66.71 (18.02–236.15)
Central Europe	30,488.91 (26,793.28 to 34,697.76)	0.00 (−0.00 to 0.00)	74.70 (22.49–248.04)	0.01 (−0.06 to 0.04)	74.67 (22.43–248.23)
Eastern Europe	31,243.12 (27,766.56 to 34,948.02)	0.00 (−0.02 to 0.02)	96.83 (33.12–286.15)	0.00 (−0.03 to 0.03)	96.81 (33.07–286.63)
High-income	32,202.15 (28,549.08 to 36,321.00)	−0.00 (−0.02 to 0.01)	68.85 (18.18–247.13)	−0.00 (−0.05 to 0.02)	68.81 (18.12–247.70)
Australasia	27,215.52 (23,764.26 to 31,068.68)	−0.00 (−0.00 to 0.00)	60.99 (17.51–200.38)	0.01 (−0.07 to 0.08)	60.92 (17.49–200.55)
High-income Asia Pacific	29,867.61 (26,437.50 to 33,548.63)	0.01 (−0.02 to 0.04)	63.72 (17.35–216.52)	0.00 (−0.04 to 0.05)	63.70 (17.36–217.39)
High-income North America	34,180.26 (30,603.85 to 38,251.42)	−0.02 (−0.05 to 0.00)	70.01 (17.89–257.69)	−0.02 (−0.06 to 0.02)	69.93 (17.83–257.73)
Southern Latin America	26,264.27 (22,771.94 to 30,205.10)	0.01 (−0.00 to 0.02)	59.96 (16.44–196.75)	0.00 (−0.08 to 0.08)	59.83 (16.38–196.27)
Western Europe	32,770.54 (28,999.01 to 37,028.00)	0.00 (−0.01 to 0.01)	71.93 (19.05–261.97)	0.00 (−0.06 to 0.04)	71.74 (18.95–261.72)
Latin America and Caribbean	25,685.02 (22,480.17 to 29,170.88)	−0.00 (−0.01 to 0.01)	53.59 (15.02–185.16)	0.00 (−0.04 to 0.04)	53.44 (14.97–185.27)
Andean Latin America	20,715.91 (17,622.56 to 23,911.58)	0.01 (−0.02 to 0.05)	48.09 (14.42–173.97)	0.01 (−0.08 to 0.11)	47.90 (14.36–173.69)
Caribbean	23,730.24 (20,293.00 to 27,687.86)	−0.00 (−0.00 to 0.00)	51.21 (14.83–177.77)	−0.00 (−0.05 to 0.05)	51.09 (14.78–178.75)
Central Latin America	24,421.55 (21,365.98 to 27,955.62)	−0.00 (−0.00 to 0.00)	53.44 (15.36–180.62)	0.01 (−0.04 to 0.05)	53.39 (15.30–180.96)
Tropical Latin America	29,005.61 (25,648.55 to 32,612.92)	0.00 (−0.02 to 0.02)	55.97 (14.51–205.79)	0.01 (−0.05 to 0.05)	55.73 (14.42–205.01)
Sub-Saharan Africa	22,454.11 (19,657.92 to 25,642.78)	−0.01 (−0.01 to −0.00)	53.56 (16.42–172.25)	0.00 (−0.04 to 0.04)	53.31 (16.34–173.23)
Central Sub-Saharan Africa	23,232.00 (20,084.38 to 27,079.59)	−0.00 (−0.00 to 0.00)	53.98 (16.14–173.91)	0.01 (−0.06 to 0.08)	53.98 (16.15–174.07)
Eastern Sub-Saharan Africa	18,801.27 (16,305.57 to 21,690.09)	−0.02 (−0.03 to −0.01)	48.12 (15.48–151.06)	−0.01 (−0.05 to 0.04)	47.88 (15.42–151.05)
Southern Sub-Saharan Africa	24,193.08 (21,262.85 to 27,462.85)	−0.00 (−0.00 to 0.00)	57.04 (17.38–179.73)	−0.01 (−0.05 to 0.03)	56.75 (17.19–179.38)
Western Sub-Saharan Africa	25,108.38 (22,066.05 to 28,468.24)	0.00 (−0.00 to 0.00)	57.49 (17.21–191.19)	0.02 (−0.04 to 0.06)	57.38 (17.21–191.73)
North Africa and Middle East	24,090.29 (20,941.08 to 27,533.32)	0.01 (−0.00 to 0.02)	67.78 (22.25–202.06)	0.00 (−0.09 to 0.06)	67.63 (22.16–202.16)
Southeast Asia	25,256.68 (22,085.09 to 28,547.33)	0.00 (−0.00 to 0.00)	51.03 (13.89–181.26)	0.01 (−0.04 to 0.06)	51.15 (13.92–181.17)

Abbreviation: GBD, Global Burden of Diseases, Injuries, and Risk Factors; TTH, tension-type headaches; UI, uncertainty interval; YLDs, years lived with disability.

**Figure 1:**
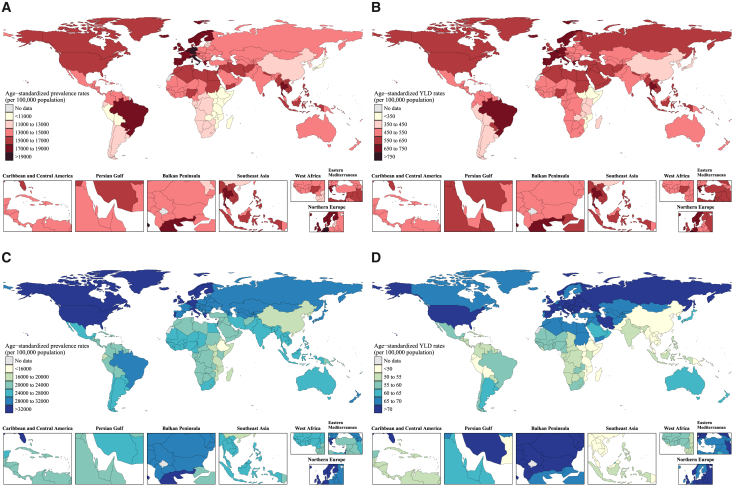
World map of age-standardized prevalence and YLD rates of migraine and TTH in 2021 (A–D) (A) and (B) represent the 2021 values of age-standardized prevalence and YLD rates for migraine, respectively, while (C) and (D) represent the 2021 values of age-standardized prevalence and YLD rates for TTH, respectively. Abbreviation: TTH; tension-type headaches; YLDs, years lived with disability.

There are geographical differences in age-standardized migraine prevalence (Table S1; Figure 1A), ranging from the lowest in Ethiopia (8,365.4 per 100,000 [95% UI, 7,232.9–9,566.2]) to the highest in Belgium (21,751.5 per 100,000 [18,730.1–25,705.5]), and YLD (Table S1; Figure 1B), ranging from the lowest in Ethiopia (313.6 per 100,000 [64.0–683.8]) to the highest in Belgium (800.4 per 100,000 [92.0–1772.0]). Overall, among 21 GBD regions, Tropical Latin America and Western Europe showed the highest burden of migraine, and Eastern Sub-Saharan Africa had the lowest, as measured by age-standardized prevalence and YLD rates. In addition, regional incidence estimates of migraine in 2021 and changes since 1990 are provided in Table S1.

### Global, regional, and national burden of TTH, 1990–2021

The number of TTH cases in 2021 was 2.0 billion (95% UI, 1.8–2.3) globally, representing a 56.4% rise from 1990 (1.3 billion [1.1–1.5]). However, the global age-standardized prevalence rate of TTH remained stable (24,904.9 per 100,000 in 1990; 24,764.8 per 100,000 in 2021; Table 1). Similarly, the YLD count for TTH in 2021 was 4.6 million (95% UI, 1.3–15.0), representing a 61.4% increase from 1990, when the YLD count was 2.8 million (0.8–9.6). The age-standardized YLD rate remained stable, from 57.0 per 100,000 in 1990 to 55.7 per 100,000 in 2021 (Table 1).

We observed national disparities in the burden of TTH (Table S2; Figures 1C and 1D), with an age-standardized prevalence rate ranging from 15,855.3 per 100,000 (95% UI, 13,637.6–18,206.9) in Ethiopia to 35,492.4 per 100,000 (31,718.0–39,481.9) in Norway and age-standardized YLD rate ranging from 40.3 per 100,000 (13.0–128.5) in Ethiopia to 99.7 per 100,000 (34.8–288.4) in Russian Federation. Regionally, as measured by age-standardized prevalence and YLD rates, the most significant burden was observed in high-income North America, while the lowest burden was in East Asia. Regional estimates of TTH incidence in 2021, along with the percentage change from 1990 to 2021, are presented in Table S2.

### Burden of headaches according to age and sex

**Figure 2:**
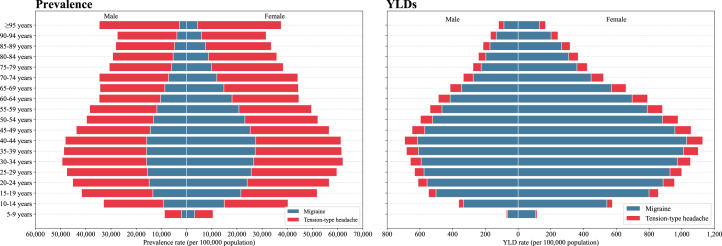
Prevalence and YLD of migraine and TTH by age group and sex in 2021 Abbreviation: TTH, tension-type headaches; YLDs, years lived with disability.

Figure 2 illustrates age- and sex-specific prevalence and YLD rates for headache disorders in 2021. Migraines and TTH were more prevalent in females than males across all age groups, with TTH showing consistently higher prevalence rates than migraines in all demographic groups. The age-specific prevalence of both conditions was highest during young-to-middle adulthood, peaking between the ages of 30 and 44 years. Notably, while the prevalence of migraines declined steadily with advancing age, TTH showed greater persistence in older age groups, remaining relatively prevalent even among old adults.

The age-specific YLD rates for migraines and TTH largely mirrored the age-specific prevalence patterns, with females experiencing a notably greater disability burden than males across all age groups. The highest age-specific YLD rates for both conditions were observed in the 40–49 age group in both sexes. However, unlike prevalence, migraines consistently exhibited higher YLD rates than TTH across all age and sex groups.

### Burden of headache according to SDI and HAQ Index

**Figure 3:**
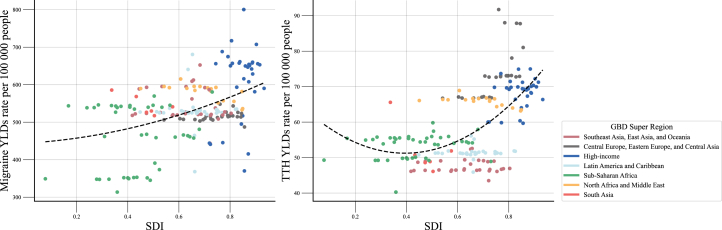
Age-standardized YLD rates of migraine and TTH by SDI level Abbreviation: GBD, Global Burden of Diseases, Injuries, and Risk Factors; SDI, Socio-demographic Index; TTH, tension-type headaches; YLDs, years lived with disability.

Figure 3 presents age-standardized YLD rates for migraine and TTH according to SDI levels in 2021. Higher age-standardized YLD rates of migraine were observed in countries with higher SDI. However, for TTH, while countries with high SDI had the highest age-standardized YLD rates, the lowest rates were observed in middle SDI. A similar pattern was observed for the HAQ Index (Figures S1 and S2).

When stratified by five SDI levels, high-SDI regions had the highest age-standardized prevalence (15,365.1 per 100,000 [95% UI, 13,250.3–17,765.4]) and YLD rates (573.6 per 100,000 [98.8–1236.0]) for migraines, while low-SDI regions had the lowest age-standardized prevalence (12,809.0 per 100,000 [10,909.3–14,754.4]) and YLD rates (475.2 per 100,000 [76.4–1031.5]; Figure S3). Similarly, for TTH, high-SDI regions had the highest age-standardized prevalence (30,603.3 per 100,000 [95% UI, 27,152.9–34,513.4]) and YLD rates (66.8 per 100,000 [18.1–235.8]). The age-standardized prevalence for TTH was lowest in the low-SDI region (22,780.2 per 100,000 [95% UI, 19,950.9–25,943.9]); however, the YLD rate was lowest in the middle-SDI region (51.5 per 100,000 [15.0–174.8]).

### Headache burden in the future up to 2050

**Figure 4:**
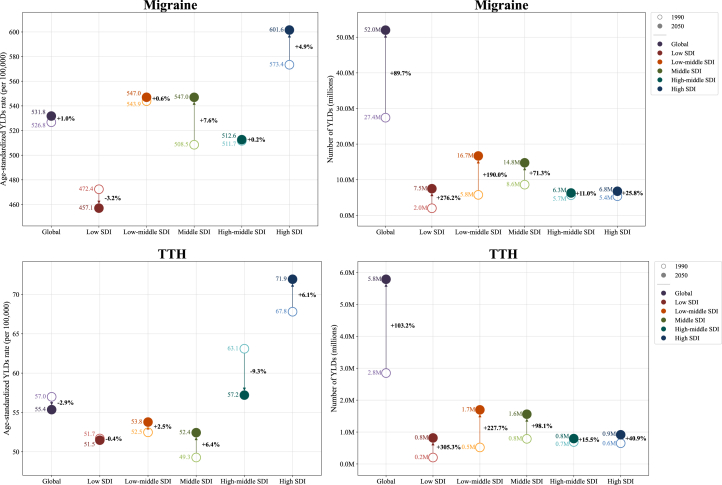
Projected YLD counts and age-standardized rates of migraine and TTH until 2050 by SDI level Abbreviation: SDI, Socio-demographic Index; TTH, tension-type headaches; YLDs, years lived with disability.

The global number of YLDs attributable to migraine is projected to increase from 43.4 million (95% UI, 6.7–95.1) in 2021 to 52.0 million (8.7–111.6) by 2050. Despite this increase, the age-standardized YLD rates for migraines are anticipated to remain stable, with estimates of 531.8 per 100,000 (95% UI, 81.1–1,165.4) in 2050 compared to 532.7 per 100,000 (80.6–1,167.7) in 2021 (Table 1). As presented in Figure 4, similar findings are observed across all five SDI levels; the highest age-standardized rates of migraine in 2050, similar to 1990, will be observed in high-SDI countries (601.6 per 100,000 [95% UI, 100.9–1,290.9]), and the lowest in low-SDI countries (457.1 per 100,000 [79.3–985.2]).

**Figure 5:**
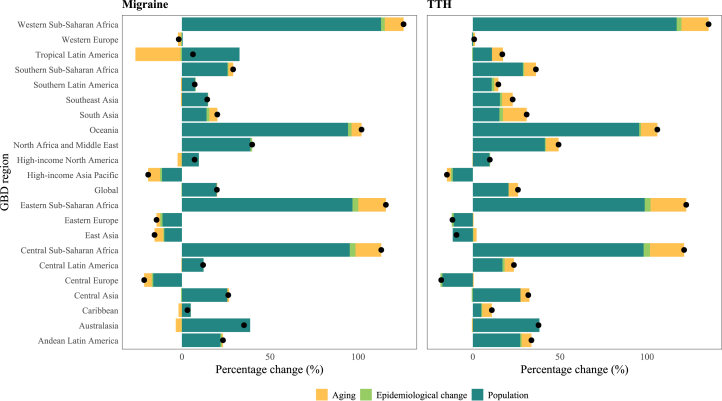
Decomposition analysis of percentage change in the number of YLDs due to migraine and TTH between 2021 and 2050, globally and by GBD region Abbreviation: GBD, Global Burden of Diseases, Injuries, and Risk Factors; TTH, tension-type headaches; YLDs, years lived with disability.

Similarly, the number of YLDs due to TTH is expected to rise from 4.6 million (95% UI, 1.3–15.0) in 2021 to 5.8 million YLDs (1.8–18.1) in 2050. However, the age-standardized YLD rates for TTH are also expected to remain stable, with estimates of 531.8 (95% UI, 81.1–1,165.4) per 100,000 in 2050 and 532.7 per 100,000 (80.6–1,167.7) in 2021 (Table 1). In 2050, the highest age-standardized rates of TTH will be found in high-SDI countries (71.9 per 100,000 [95% UI, 20.4–247.9]), while the lowest will be found in low-SDI countries (51.5 per 100,000 [15.7–167.1]). A detailed depiction of the percentage changes in both age-standardized YLD rates and the number of YLDs for migraine and TTH from 1990 to 2050 is provided in Figure 4. At the regional level, the age-standardized YLD rates for migraine and TTH are expected to change minimally. However, in some regions, the rates are expected to decrease, including Central Europe (−21.4%), high-income Asia Pacific (−19.2%), East Asia (−15.5%), and Eastern Europe (−14.4%). Our decomposition analysis, both regionally and globally, revealed that population growth will be the primary driver of the projected change in the number of YLDs by 2050 (Figure 5), a trend observed across all regions.

## Discussion

This study provides a comprehensive assessment of the global, regional, and national burden of headache disorders, including migraines and TTH, from 1990 to 2021, and the forecasts up to 2050 based on GBD 2021 estimates. In 2021, TTH was the second most common specific condition, while migraine was the seventh. While the absolute number of migraine and TTH cases increased, age-standardized prevalence and YLD rates remained stable or had a minimal increase. We found that the age-standardized disability burden of migraine and TTH is forecasted to remain stable up to 2050, driven by population growth and demographic changes rather than worsening individual outcomes, highlighting the importance of sustained healthcare access and effective management strategies to reduce the burden.

Although TTH was more prevalent than migraine, migraine accounted for a disproportionately higher disability burden, reflecting its greater impact on quality of life. In addition, there were substantial variations in the burden of these conditions across regions and demographic groups in 2021. Countries with higher socioeconomic development and better healthcare access (i.e., countries with high SDI and HAQ Index) tend to have a higher burden of headache disorders, as measured by age-standardized prevalence and YLD rates, which could be due to a combination of better diagnosis and modern lifestyle risk factors. In addition, young and middle-aged females will be disproportionately affected by headache disorders, underscoring the significance of addressing the unique needs of females suffering from headache disorders.

The stability of age-standardized estimates for headache disorders, including migraines and TTH, observed in GBD 2021, can be attributed to a combination of persistent risk factors and improvements in healthcare systems. Stress, sedentary lifestyles, caffeinated or alcohol consumption, and poor-quality sleep remain widespread triggers, sustaining the burden of these conditions globally.^12^ While advancements in diagnostic tools and treatment options have improved the recognition and management of headache disorders, they have primarily mitigated underreporting.^13^ Looking toward 2050, these rates are expected to remain stable due to the enduring influence of these modern risk factors. Furthermore, the limited utilization of professional healthcare for headaches, with many individuals relying on over-the-counter remedies,^14^ may continue to impede substantial reductions in prevalence and disability. This equilibrium suggests that healthcare advancements balance but do not eliminate the underlying contributors to headache disorders, leaving the burden relatively stable.

We also found that higher age-standardized prevalence and YLD rates for migraines and TTHs were generally observed in high SDI and HAQ Index, and this trend is expected to continue up to 2050. This may be due to modern lifestyle factors, such as chronic psychosocial stress, high occupational demands, sedentary behavior, higher diagnostic capacity, heightened health literacy, and widespread access to healthcare services.^15^ Conversely, low-SDI regions appear to have a lower burden, possibly due to a combination of underdiagnosis, limited access to healthcare professionals trained in headache disorders, and overall underreporting.^16^^,^^17^ These patterns suggest that tailored approaches are needed—focusing on improving healthcare access and reducing lifestyle-related risk factors in high-SDI regions while addressing healthcare gaps in low-SDI areas.

Headache disorders disproportionately affect women, particularly in young and middle-aged groups, which can be explained by a complex interplay of biological, hormonal, environmental, and psychological factors. Hormonal fluctuations, particularly changes in estrogen levels during the menstrual cycle, pregnancy, and menopause, could play an essential role in the pathophysiology of migraines.^18^ These fluctuations, combined with the demands of balancing family, career, and social responsibilities, may contribute to the higher prevalence of headache disorders in women.^18^ In addition, psychological conditions such as anxiety and depression, which are more common in women,^19^ could exacerbate the frequency and severity of migraines and TTH. Furthermore, the larger gap observed in YLD rates compared to prevalence between sexes suggests that these risk factors may not only increase the occurrence of headaches in women but could also intensify their associated disability and impact on daily life.

While TTH is more prevalent than migraines across all demographic groups, migraines consistently exhibited higher YLD rates, highlighting their greater severity and disabling nature. These headaches often impair daily functioning and significantly reduce quality of life.^20^^,^^21^ Conversely, TTH, although more common, typically results in milder symptoms and a lesser degree of disability. This divergence underscores the need for tailored management strategies: addressing the debilitating effects of migraines through comprehensive treatment and support while implementing preventive measures for the broader but less severe impact of TTH.^4^

While the burden of many fatal and disabling disorders diminished with socioeconomic development,^22^^,^^23^ that of migraines and TTHs remain persistent, underscoring the need for continued and targeted public health efforts for disproportionated demographic groups. Despite being a major cause of disability, headache disorders are often overlooked in public health strategies, research, and training programs.^24^ For instance, less than 30% of primary care physicians in the United States were aware of the American Academy of Neurology’s migraine prevention guidelines and important risk factors.^25^ Females, particularly in their most productive years, experience the highest prevalence and YLD rates, exacerbating the societal and economic impact of these conditions. This underscores the need for public health initiatives that focus on tailored interventions, such as education, early diagnosis, and sex-sensitive healthcare policies, to reduce the long-term effects on females, especially addressing the unique psychosocial challenges they face.^26^

In addition, disparities between countries with different SDI levels further complicate headache care. High-SDI countries tend to report a higher burden of headaches, with better diagnosis but less effective management due to lifestyle factors. In contrast, developing countries often face limited access to healthcare and essential treatments, exacerbating the global disparity. Structured healthcare systems, such as tiered headache services and education regarding the chronic nature of headache disorders, can help bridge these gaps by training primary care providers and ensuring that even underserved populations can receive appropriate diagnosis and management, improving care access across diverse socioeconomic settings.^17^

The COVID-19 pandemic introduced additional challenges for headache management, with acute or chronic headaches after SARS-CoV-2 infection or vaccination adding to the global burden.^8^^,^^9^^,^^10^ Some patients with migraines co-infected with SARS-CoV-2 report experiencing more severe pain that is often less responsive to standard analgesics.^27^ These new headache issues, combined with increased psychological stress, disrupted healthcare access, and social factors, may have further complicated an already pressing public health issue. Therefore, effective management should address both the biological impacts of the virus and vaccination, as well as the psychological and social factors exacerbating these conditions.^27^

### Conclusion

Based on the findings of GBD 2021 estimates, headache disorders, including migraines and TTH, remain an immense global health burden, with a disproportionate burden in young to middle-aged females. The burden of headache disorders also substantially varies across SDIs and HAQ Index, with high-SDI and high-HAQ Index regions facing higher prevalence and disability rates due to modern lifestyle factors, while low-SDI regions experience underreporting and limited healthcare access. Region-specific strategies are necessary to address this, focusing on improving healthcare access and affordability of treatment in low-SDI areas. Tailored interventions and resource allocation can reduce the burden of these disorders moving toward 2050.

### Limitations of the study

This study provided updated GBD 2021 analyses of TTH and migraine burden for 1990 to 2021, with the headache disability burden forecasts up to 2050. However, several limitations should be addressed in this study. Our study faces limitations in all GBD works, including the lack of reliable epidemiological data in low- and middle-income countries. Headache disorders are often inadequately represented in national health surveys, and administrative data sources, such as claims or primary care records, frequently fail to capture their prevalence reliably. As a result, country-specific estimates rely on predictive covariates and data from neighboring countries, resulting in greater UIs in these settings. Although the GBD framework employs statistical techniques such as hierarchical modeling and cross-location information borrowing to mitigate data sparsity, region-specific sensitivity analyses or uncertainty decompositions were not conducted. Therefore, residual uncertainty may be underestimated in regions with severely limited input data, and estimates from these areas should be interpreted with caution. Second, limitations in data collection and analysis methods prevented the ability to distinguish between probable and definite diagnoses of migraine based on the International Classification of Headache Disorders (ICHD) criteria. However, it is important to note that probable migraine carries a substantial disease burden and disability similar to that of definite migraine.^28^^,^^29^ The primary issue leading to a probable rather than definite diagnosis in past studies has often been the duration of the headache. Therefore, the GBD study’s approach of categorizing probable migraine within the broader category of migraine may be reasonable. This method may ensure a comprehensive understanding of the disease burden. Third, a similar argument can be made for probable TTH, although the knowledge of this type of headache is significantly less developed. Additionally, the YLDs missed by excluding this headache type are fewer due to the considerably lower disability weight (DW) associated with TTH compared to migraine.^1^ Fourth, since the GBD estimates are limited to 2021, we did not conduct secondary analyses to evaluate the impact of the COVID-19 pandemic on headache disorders. The GBD 2021 estimates and forecasts did not account for the effects of the pandemic or recent conflicts on headache burden, as geographically and temporally comprehensive data were unavailable at the time of analysis. We aim to explore the impact of COVID-19 on headaches in future studies as more robust and diverse data become accessible. Fifth, we did not quantify the burden of headache disorders attributable to any risk factors, such as diet, psychological factors, and environmental triggers.^13^ Nonetheless, further research is necessary to better understand the impact of these risk factors on headache disorder burden and to refine data collection methods for more accurate assessments in future studies. Finally, our projections were based solely on the SDI as the predictive covariate. While SDI captures broad socioeconomic patterns, it may not adequately account for other influential factors, such as healthcare accessibility, evolving diagnostic practices, and environmental exposures.

## Resource availability

### Lead contact

Requests for additional information, resources, and reagents should be directed to and will be fulfilled by the lead contact, Dong Keon Yon (yonkkang@gmail.com).

### Materials availability

No new unique materials were generated in this study.

### Data and code availability

• The data used in this study are publicly available through the Global Health Data Exchange (GHDx) GBD 2021 website: https://ghdx.healthdata.org/gbd-2021.
• This study did not generate any original code.
• Any additional information required to reanalyze the data reported in this paper is available from the lead contact upon request.

## Consortia

The members of the GBD 2021 Headache Collaborators are Tissa Wijeratne, Jiyeon Oh, Soeun Kim, Yesol Yim, Min Seo Kim, Jae Il Shin, Yun-Seo Oh, Raon Jung, Yun Seo Kim, Lee Smith, Hasan Aalruz, Rami Abd-Rabu, Deldar Morad Abdulah, Richard Gyan Aboagye, Meysam Abolmaali, Dariush Abtahi, Ahmed Abualhasan, Rufus Adesoji Adedoyin, Qorinah Estiningtyas Sakilah Adnani, Fatemeh Afrashteh, Navidha Aggarwal, Danish Ahmad, Ali Ahmadi, Negar Sadat Ahmadi, Amir Mahmoud Ahmadzade, Syed Anees Ahmed, Salah Al Awaidy, Sawsan Alabbad, Muaaz M. Alajlani, Yazan Al-Ajlouni, Mohammed Usman Ali, Syed Shujait Ali, Waad Ali, Joseph Uy Almazan, Najim Z. Alshahrani, Awais Altaf, Mohammad Al-Wardat, Karem H. Alzoubi, Sohrab Amiri, Hubert Amu, Ganiyu Adeniyi Amusa, David B. Anderson, Saleha Anwar, Demelash Areda, Mohammad Asghari-Jafarabadi, Sait Ashina, Javed Ashraf, Tahira Ashraf, Ali Azargoonjahromi, Yogesh Bahurupi, Atif Amin Baig, Soham Bandyopadhyay, Mainak Bardhan, Hiba Jawdat Barqawi, Azadeh Bashiri, Mohammad-Mahdi Bastan, Maryam Bemanalizadeh, Isabela M. Bensenor, Alemshet Yirga Yirga Berhie, Akshaya Srikanth Bhagavathula, Sonu Bhaskar, Vivek Bhat, Gurjit Kaur Bhatti, Jasvinder Singh Bhatti, Cem Bilgin, Atanu Biswas, Bruno Bizzozero-Peroni, Yasser Bustanji, Luis Alberto Cámera, Edoardo Caronna, Andre F. Carvalho, Sandip Chakraborty, Patrick R. Ching, Nikos Christodoulou, Dinh-Toi Chu, Hongyuan Chu, Natalia Cruz-Martins, Omid Dadras, Xiaochen Dai, Emanuele D'Amico, Amira Hamed Darwish, Sindhura Deekonda, Vinoth Gnana Chellaiyan Devanbu, Samath Dhamminda Dharmaratne, Adriana Dima, Temesgien Ergetie Dinkayehu, Huyen Do, Paul Narh Doku, Ojas Prakashbhai Doshi, Abdel Rahman E’mar, Negin Eissazade, Chadi Eltaha, Ayesha Fahim, Jawad Fares, Mohsen Farjoud Kouhanjani, Andre Faro, Patrick Fazeli, Seyed-Mohammad Fereshtehnejad, Pietro Ferrara, Nuno Ferreira, Florian Fischer, Arianna Fornari, Márió Gajdács, Miglas Welay Gebregergis, Delaram J. Ghadimi, Amir Ghaffari Jolfayi, Elena V. Gnedovskaya, Mahaveer Golechha, Enrique Gomez Figueroa, Mohammad Hashem Hashempur, Md Saquib Hasnain, Amr Hassan, Nageeb Hassan, Mahgol Sadat Hassan Zadeh Tabatabaei, Mohamed I. Hegazy, Golnaz Heidari, Bartosz Helfer, Md Mahbub Hossain, Mowafa Househ, Chengxi Hu, Ivo Iavicoli, Olayinka Stephen Ilesanmi, Irena M. Ilic, Muhana Fawwazy Ilyas, Salim Ilyasu, Nahlah Elkudssiah Ismail, Ali Jafari-Khounigh, Haitham Jahrami, Manthan Dilipkumar Janodia, Ruwan Duminda Jayasinghe, Bijay Mukesh Jeswani, Jost B. Jonas, Nitin Joseph, Rizwan Kalani, Moien A.B. Khan, Sorour Khateri, Mahalaqua Nazli Khatib, Hamid Reza Khayat Kashani, Feriha Fatima Khidri, Moein Khormali, Sepehr Khosravi, Yun Jin Kim, Farzad Kompani, Karel Kostev, Kewal Krishan, Bindu Krishnan, Barthelemy Kuate Defo, Mohammed Kuddus, Mukhtar Kulimbet, Rakesh Kumar, Vijay Kumar, Ville Kytö, Savita Lasrado, Seung Won Lee, Jacopo Lenzi, Matilde Leonardi, Giancarlo Lucchetti, Alessandra Lugo, Irsa Fatima Makhdoom, Arashk Mallahzadeh, Vahid Mansouri, Roy Rillera Marzo, Yasith Mathangasinghe, Mahsa Mayeli, Asim Mehmood, Atte Meretoja, Tomislav Mestrovic, Sachith Mettananda, Giuseppe Minervini, Archana Mishra, Prasanna Mithra, Khabab Abbasher Hussien Mohamed Ahmed, Ibrahim Mohammadzadeh, Shafiu Mohammed, Lorenzo Monasta, Shane Douglas Morrison, Amin Nabavi, Zuhair S. Natto, Javaid Nauman, Luciano Nieddu, Fred Nugen, Andrew T. Olagunju, Arão Belitardo Oliveira, Welber Sousa Oliveira, Hany A. Omar, Goran Latif Omer, Nikita Otstavnov, Mahesh P.A., Leonidas D. Panos, Romil R. Parikh, Shankargouda Patil, Apurba Patra, Paolo Pedersini, Umberto Pensato, Prince Peprah, Mario F.P. Peres, Michael A. Piradov, Patricia Pozo-Rosich, Jalandhar Pradhan, Sanjay Prakash, Akila Prashant, Jagadeesh Puvvula, Alireza Rafiei, Alberto Raggi, Amir Masoud Rahmani, Mahmoud Mohammed Ramadan, Devarajan Rathish, Ilari Rautalin, Salman Rawaf, Mohsen Rezaeian, Donya Rezazadeh Eidgahi, Taeho Gregory Rhee, Priyanka Roy, Adnan Saad Eddin, Cameron John Sabet, Basema Ahmad Saddik, Erfan Sadeghi, Umar Saeed, Fatemeh Saheb Sharif-Askari, Narjes Saheb Sharif-Askari, Pragyan Monalisa Sahoo, Sohrab Salimi, Abdallah M. Samy, Jennifer Saulam, Monika Sawhney, Yigit Can Senol, Subramanian Senthilkumaran, Yashendra Sethi, Homa Seyedmirzaei, Mahan Shafie, Anas Shamsi, Amin Sharifan, Hatem Samir Shehata, Rekha Raghuveer Shenoy, Farhad Shokraneh, Jaspreet Kaur Sidhu, Baljinder Singh, Harmanjit Singh, Jasvinder A. Singh, Surjit Singh, Anna Aleksandrovna Skryabina, Farrukh Sobia, Bahadar S. Srichawla, Vinay Suresh, Chandan Kumar Swain, Sree Sudha T.Y., Payam Tabaee Damavandi, Celine Tabche, Mohammad Tabish, Manoj Tanwar, Mohamad-Hani Temsah, Masayuki Teramoto, Nghia Minh Tran, Thang Huu Tran, Aristidis Tsatsakis, Aniefiok John Udoakang, Jibrin Sammani Usman, Hande Uzunçıbuk, Jef Van den Eynde, Tommi Juhani Vasankari, Narayanaswamy Venketasubramanian, Jorge Hugo Villafañe, Lintao Wang, Xingxin Wang, Yuan-Pang Wang, Taweewat Wiangkham, Andrea Sylvia Winkler, Alemayehu Molla Wollie, Zheman Xiao, Yazachew Engida Engida Yismaw, Abdilahi Yousuf, Zhongyi Zhao, Magdalena Zielińska, Min Kyung Chu, Tae-Jin Song, Dong Keon Yon, and Valery L. Feigin.

## Acknowledgments

This study was funded by the 10.13039/100000865Bill and Melinda Gates Foundation (OPP1152504); the Australian National Health and Medical Research Council; and the Queensland Department of Health, Australia. The Yonsei Fellowship, funded by Youn Jae Lee (to J.I.S.), also supported this work. This work was supported by the Institute of Information & Communications Technology Planning & Evaluation (IITP) grant funded by the Korean government (RS-2024-00509257 [Global AI Frontier Lab] to D.K.Y.). This work was supported by the Institute of Information & communications Technology Planning & Evaluation (IITP) grant funded by the Korean government (2022-0-00621 or RS-2022-II220621 [Development of artificial intelligence technology that provides dialog-based multi-modal explainability] to T.J.S.). This research was supported by a grant from the Korea Health Technology R&D Project through the 10.13039/501100003710Korea Health Industry Development Institute (KHIDI), funded by the Ministry of Health & Welfare, Republic of Korea (RS-2023-00262087 to T.J.S. and RS-2025-02220492 to D.K.Y.). This research was supported by a grant from the 10.13039/501100003725National Research Foundation of Korea (NRF), funded by the Korean government (MSIT) (2022R1A2C1091767 to M.K.C.). This research was supported by the BK21 FOUR (Fostering Outstanding Universities for Research) funded by the Ministry of Education (MOE, Korea) and National Research Foundation of Korea (NRF-5199990614253 to T.-J.S., Education Research Center for 4IR-Based Health Care). The study’s funder had no role in the study design, data collection, data analysis, data interpretation, or writing of the report. All authors had full access to the study data and had final responsibility for the decision to submit the manuscript for publication.

## Author contributions

Members of the core research team for this topic area had full access to the underlying data used to generate estimates presented in this study. All other authors had access to and reviewed estimates as part of the research evaluation process, which includes additional stages of formal review. Conceptualization and design: T. Wijeratne, J.O., S. Kim, Y.Y., M.S.K., J.I.S., M.K.C., T.-J.S., D.K.Y., and V.F.; methodology: T. Wijeratne, J.O., S. Kim, Y.Y., M.S.K., J.I.S., M.K.C., T.-J.S., D.K.Y., and V.F.; data acquisition: T. Wijeratne, J.O., S. Kim, Y.Y., M.S.K., J.I.S., M.K.C., T.-J.S., D.K.Y., and V.F.; statistical analysis and data curation: T. Wijeratne, J.O., S. Kim, Y.Y., M.S.K., J.I.S., M.K.C., T.-J.S., D.K.Y., and V.F.; validation: D.K.Y. and V.F.; data interpretation: D.K.Y. and V.F.; visualization: D.K.Y. and V.F.; managing the estimation or publications process: D.K.Y. and V.F.; writing – original draft preparation: T. Wijeratne, J.O., S. Kim, and Y.Y.; writing – review and editing: all authors; supervision: M.K.C., T.-J.S., and D.K.Y.; project administration: M.K.C., T.-J.S., and D.K.Y.; funding acquisition: M.K.C., T.-J.S., and D.K.Y. T. Wijeratne, J.O., S. Kim, Y.Y., M.S.K., and J.I.S. contributed equally as the first authors. M.K.C., T.-J.S., and D.K.Y. contributed equally as corresponding authors. V.F. is the senior author. The corresponding authors and the senior author had full access to the data in the study and had final responsibility for the decision to submit for publication. Author contributions by the GBD 2021 Global Headache Collaborators are shown in the following.

Managing the estimation or publications process: D.K.Y. and M.S.K..

Providing data or critical feedback on data sources: R.A.-R., R.G.A., A. Abualhasan, Q.E.S.A., D. Ahmad, A.A., S.A. Awaidy, S. Alabbad, M.M.A., S.S.A., J.U.A., A. Altaf, M.A.-W., H. Amu, S. Anwar, T.A., A.A.B., S. Bandyopadhyay, M. Bardhan, M.-M.B., M. Bemanalizadeh, A.S.B., S. Bhaskar, J.S.B., G.K.B., C.B., E.C., N.C., H.C., D.-T.C., N.C.-M., X.D., S.D., V.G.C.D., S.D.D., H.D., O.P.D., C.E., A. Fahim, J.F., S.-M.F., A. Fornari, M. Golechha, M.S.H.Z.T., M.I.H., G.H., M.M.H., M.H., C.H., O.S.I., M.F.I., N.E.I., H.J., B.M.J., J.B.J., M.A.B.K., S. Khateri, F.F.K., Y.J.K., K. Krishan, B.K.D., V. Kumar, V. Kytö, S.L., S.W.L., R.R.M., A. Mehmood, A. Meretoja, S. Mettananda, A. Mishra, K.A.H.M.A., S. Mohammed, L.M., Z.S.N., L.N., F.N., A.T.O., W.S.O., A.B.O., H.A.O., M.P.A., L.D.P., R.R.P., S. Patil, A. Patra, P. Peprah, M.F.P.P., J. Pradhan, S. Prakash, J. Puvvula, A.M.R., M.M.R., I.R., S.R., T.G.R., P.R., A.S.E., C.J.S., B.A.S., U.S., N.S.S.-A., P.M.S., A.M.S., M. Sawhney, Y.C.S., S. Senthilkumaran, Y.S., H. Seyedmirzaei, A. Shamsi, A. Sharifan, J.I.S., F. Shokraneh, B.S., H. Singh, J.A.S., A.A.S., L.S., C.K.S., S.S.T.Y., C.T., M. Tanwar, J.V.D.E., T.J.V., N.V., X.W., L.W., T. Wiangkham, T. Wijeratne, Z.X., Y.E.E.Y., A.Y., Z.Z., and M.Z.

Developing methods or computational machinery: X.D. and L.S.

Providing critical feedback on methods or results: D.M.A., R.G.A., M.A., D. Abtahi, A. Abualhasan, Q.E.S.A., F.A., N.A., D. Ahmad, A. A., N.S.A., A.M.A., S.A. Ahmed, S.A. Awaidy, S. Alabbad, M.M.A., M.U.A., W.A., S.S.A., J.U.A., N.Z.A., A. Altaf, M.A.-W., K.H.A., S. Amiri, H. Amu, G.A.A., D.B.A., S. Anwar, D. Areda, M.A.-J., S. Ashina, J.A., T.A., A. Azargoonjahromi, Y. Bahurupi, A.A.B., S. Bandyopadhyay, M. Bardhan, H.J.B., M.-M.B., M. Bemanalizadeh, I.M.B., A.Y.Y.B., A.S.B., S. Bhaskar, V.B., J.S.B., G.K.B., C.B., B.B.-P., Y. Bustanji, L.A.C., A.F.C., N.C., H.C., D.-T.C., N.C.-M., O.D., X.D., A.H.D., S.D., V.G.C.D., S.D.D., A.D., T.E.D., H.D., P.N.D., O.P.D., A.R.E., N.E., C.E., A. Fahim, J.F., M.F.K., A. Faro, P. Fazeli, S.-M.F., F.F., A. Fornari, M. Gajdács, M.W.G., D.J.G., A.G.J., E.V.G., M. Golechha, E.G.F., M.H.H., M.S.H., M.S.H.Z.T., G.H., B.H., M.M.H., M.H., C.H., O.S.I., I.M.I., M.F.I., S.I., N.E.I., A.J.-K., H.J., R.D.J., B.M.J., J.B.J., N.J., M.A.B.K., S. Khateri, M.N.K., F.F.K., M. Khormali, M.S.K., Y.J.K., F.K., K. Krishan, B.K.D., M. Kuddus, V. Kumar, V. Kytö, S.L., S.W.L., J.L., M.L., G.L., I.F.M., A. Mallahzadeh, V.M., R.R.M., Y.M., M.M., A. Mehmood, A. Meretoja, T.M., S. Mettananda, G.M., A. Mishra, P.M., K.A.H.M.A., I.M., S. Mohammed, A.N., Z.S.N., J.N., L.N., F.N., A.T.O., A.B.O., H.A.O., G.L.O., N.O., M.P.A., L.D.P., R.R.P., S. Patil, A. Patra, P. Pedersini, P. Peprah, M.F.P.P., M.A.P., P.P.-R., J. Pradhan, S. Prakash, A. Prashant, J. Puvvula, A. Rafiei, A. Raggi, A.M.R., M.M.R., D.R., I.R., S.R., M.R., T.G.R., P.R., A.S.E., C.J.S., B.A.S., E.S., U.S., N.S.S.-A., F.S.S.-A., P.M.S., S. Salimi, A.M.S., J.S., M. Sawhney, Y.C.S., S. Senthilkumaran, Y.S., H. Seyedmirzaei, M. Shafie, A. Shamsi, A. Sharifan, H.S.S., R.R.S., J.I.S., F. Shokraneh, J.K.S., B.S., H. Singh, J.A.S., A.A.S., L.S., V.S., C.K.S., S.S.T.Y., P.T.D., C.T., M. Tabish, M. Tanwar, M.-H.T., M. Teramoto, N.M.T., A.J.U., J.S.U., H.U., J.V.D.E., N.V., J.H.V., X.W., Y.-P.W., L.W., T. Wiangkham, T. Wijeratne, A.S.W., A.M.W., Z.X., D.K.Y., A.Y., Z.Z., and M.Z.

Drafting the work or revising it critically for important intellectual content: H. Aalruz, R.A.-R., M.A., A. Abualhasan, R.A.A., Q.E.S.A., N.A., D. Ahmad, A. A., N.S.A., S.A. Ahmed, S.A. Awaidy, S. Alabbad, Y.A.-A., M.U.A., W.A., S.S.A., N.Z.A., A. Altaf, M.A.-W., K.H.A., S. Amiri, H. Amu, G.A.A., D.B.A., S. Anwar, A.A.B., S. Bandyopadhyay, M. Bardhan, H.J.B., A. Bashiri, M.-M.B., M. Bemanalizadeh, I.M.B., A.S.B., S. Bhaskar, V.B., J.S.B., G.K.B., A. Biswas, B.B.-P., Y. Bustanji, E.C., A.F.C., S.C., P.R.C., N.C., H.C., N.C.-M., E.D., A.H.D., S.D., S.D.D., A.D., H.D., P.N.D., O.P.D., A.R.E., N.E., C.E., A. Fahim, J.F., M.F.K., A. Faro, S.-M.F., P. Ferrara, N.F., F.F., M. Gajdács, M.W.G., D.J.G., E.V.G., M.H.H., M.S.H., N.H., A.H., M.S.H.Z.T., G.H., B.H., M.M.H., C.H., I.I., O.S.I., I.M.I., M.F.I., S.I., N.E.I., H.J., M.D.J., R.D.J., B.M.J., J.B.J., N.J., R. Kalani, M.A.B.K., S. Khateri, H.R.K.K., F.F.K., S. Khosravi, M.S.K., K. Kostev, K. Krishan, B.K., B.K.D., M. Kuddus, M. Kulimbet, R. Kumar, V. Kytö, S.L., J.L., M.L., G.L., A.L., I.F.M., A. Mallahzadeh, V.M., R.R.M., Y.M., M.M., A. Mehmood, A. Meretoja, T.M., S. Mettananda, G.M., A. Mishra, P.M., K.A.H.M.A., S. Mohammed, L.M., S.D.M., A.N., Z.S.N., J.N., L.N., F.N., A.T.O., A.B.O., H.A.O., N.O., M.P.A., L.D.P., R.R.P., S. Patil, A. Patra, P. Pedersini, U.P., M.F.P.P., M.A.P., P.P.-R., J. Pradhan, A. Prashant, J. Puvvula, A. Rafiei, A. Raggi, A.M.R., M.M.R., D.R., I.R., S.R., P.R., A.S.E., C.J.S., B.A.S., U.S., F.S.S.-A., A.M.S., Y.C.S., Y.S., H. Seyedmirzaei, M. Shafie, A. Shamsi, A. A.S., R.R.S., J.I.S., F. Shokraneh, H. Singh, J.A.S., S. Singh, A.A.S., L.S., F. Sobia, B.S.S., S.S.T.Y., P.T.D., C.T., M. Tanwar, M.-H.T., N.M.T., T.H.T., A.T., A.J.U., J.S.U., H.U., J.V.D.E., T.J.V., N.V., J.H.V., X.W., Y.-P.W., L.W., T. Wiangkham, T. Wijeratne, A.S.W., A.M.W., Z.X., D.K.Y., Z.Z., and M.Z.

## Declaration of interests

S. Ashina reports royalties or licenses from McGraw Hill/Medical; consulting fees from Lundbeck, Eli Lilly, Pfizer, AbbVie, Satsuma, Linpharma, Theranica, and Impel NeuroPharma; payment or honoraria for lectures, presentations, speakers bureaus, manuscript writing, or educational events from Lundbeck, Teva, Pfizer, AbbVie, and Eli Lilly; and leadership or fiduciary role in other board, society, committee, or advocacy group, paid or unpaid from the Board Trustee, International Headache Society and as a Member of Education Committee, International Headache Society; other financial or non-financial interests as Associate Editor of Cephalalgia, Associate Editor of Frontiers in Neurology, and Associate Editor of BMC Neurology; outside the submitted work. S. Bhaskar reports grants or contracts from Japan Society for the Promotion of Science (JSPS); Japanese Ministry of Education, Culture, Sports, Science and Technology (MEXT); Grant-in-Aid for Scientific Research (KAKENHI) (grant ID: 23KF0126); JSPS and the Australian Academy of Science; and JSPS International Fellowship (grant ID: P23712). S. Bhaskar reports leadership or fiduciary role in other board, society, committee, or advocacy group, paid or unpaid as District Chair, Diversity, Equity, Inclusion & Belonging of Rotary District 9675 (Sydney, Australia); as Chair, Founding Member and Manager of the Global Health & Migration Hub Community, Global Health Hub Germany (Berlin, Germany); as Editorial Board Member of PLOS One, BMC Neurology, Frontiers in Neurology, Frontiers in Stroke, Frontiers in Public Health, Journal of Aging Research, Neurology International, Diagnostics, & BMC Medical Research Methodology; as a member of the College of Reviewers, Canadian Institutes of Health Research (CIHR), Government of Canada; as the Director of Research of World Headache Society (Bengaluru, India); as Expert Adviser/Reviewer of Cariplo Foundation (Milan, Italy); as Visiting Director of National Cerebral and Cardiovascular Center, Department of Neurology, Division of Cerebrovascular Medicine and Neurology, Suita (Osaka, Japan); as Member, Scientific Review Committee of Cardiff University Biobank (Cardiff, UK); as Chair of Rotary Reconciliation Action Plan; and as Healthcare and Medical Adviser at Japan Connect (Osaka, Japan) outside the submitted work. A. Biswas reports consulting fees from Intas Pharmaceuticals, Lupin Pharmaceuticals, Alkem Laboratories, and Torrent Pharmaceuticals, outside the submitted work. C.E. reports grants or contracts from Río Hortega grant Acción Estratégica en Salud 2017–2020 from Instituto de Salud Carlos III (CM20/00217) and the Juan Rodés fellowship, Subprograma Estatal de Incorporación de la Acción Estratégica en Salud 2023 (JR23/00065) from Instituto de Salud Carlos III; payment or honoraria for lectures, presentations, speakers bureaus, manuscript writing, or educational events from Organon, TEVA, Lilly, Dr. Reddy’s, and Novartis; and support for attending meetings and/or travel from TEVA and Lundbeck outside the submitted work. X.D. reports support for the present manuscript from the Institute for Health Metrics and Evaluation, University of Washington. A Dima reports other financial interests with the Bucharest University of Economic Studies (Romania), outside the submitted work. A. Faro reports support for the present manuscript from the National Council for Scientific and Technological Development (CNPq, Brazil), personal grant “Researcher at CNPq - Level 1B.” E.G.F. reports payment or honoraria for lectures, presentations, speakers bureaus, manuscript writing, or educational events from Biogen, Novartis, Merck, Roche, and Astra Zeneca and support for attending meetings and/or travel from Biogen, Novartis, Merck, Roche, and Astra Zeneca outside the submitted work. A.H. reports consulting fees from Novartis, Sanofi Genzyme, Biologix, Merck, Hikma Pharma, Janssen, Inspire Pharma, Future Pharma, and Elixir pharma; payment or honoraria for lectures, presentations, speakers bureaus, manuscript writing, or educational events from Novartis, Allergan, Merck, Biologix, Janssen, Roche, Sanofi Genzyme, Bayer, Hikma Pharma, Al Andalus, Chemipharm, Lundbeck, Inspire Pharma, Future Pharma and Habib Scientific Office, and Everpharma; and support for attending meetings and/or travel from Novartis, Allergan, Merck, Biologix, Roche, Sanofi Genzyme, Bayer, Hikma Pharma, Chemipharm, and Al Andalus and Clavita pharm. A.H. reports leadership or fiduciary role in other board, society, committee, or advocacy group, paid or unpaid as the Vice President of MENA headache society; Board member of Multiple Sclerosis chapter of the Egyptian Society of Neurology; Board member of head-ache chapter of the Egyptian Society of Neurology; and member of committee of Education of the international Headache Society (IHS), membership committee of IHS, and regional committee of HIS outside the submitted work. I.I. reports support for the present manuscript from the Ministry of Science, Technological Development and Innovation of the Republic of Serbia (grant no. 451-03-137/2025-03/200110). N.E.I. reports leadership or fiduciary role in other board, society, committee or advocacy group, unpaid as Bursar and Council Member of the Malaysian Academy of Pharmacy (Malaysia) and committee member of Malaysian Pharmacists Society Education Chapter Committee outside the submitted work. K. Krishan reports other, non-financial support from the UGC Centre of Advanced Study, CAS II, awarded to the Department of Anthropology, Panjab University (Chandigarh, India), outside the submitted work. M.L. reports grants or contracts from the Italian Ministry of Health for research on disorders of consciousness as well as grants from EU on NCDs and CVDs impact and support for attending meetings and/or travel as Board member of the European Academy of Neurology and of the Italian Society of neurology outside the submitted work. S. Mohammed reports support for the present manuscript from the Gates Foundation. L.M. reports support for the present manuscript from the Italian Ministry of Health (Ricerca Corrente 34/2017), payments made to the Institute for Maternal and Child Health IRCCS Burlo Garofolo. W.O. reports grants or contracts from Pfizer, Libbs, Teva, Ease Labs, and Mantecorp; payment or honoraria for lectures, presentations, speakers bureaus, manuscript writing, or educational events from Pfrizer, Libbs, Teva, Ease Labs, and Mantecorp; support for attending meetings and/or travel from Mantecorp; leadership or fiduciary role in other board, society, committee, or advocacy group, unpaid with the Public Policies Committee (Brazilian Headache Society) outside the submitted work. M.F.P.P. reports grants or contracts from Abbvie and Pfizer; consulting fees from Abbvie, Pfizer, and Teva; payment or honoraria for lectures, presentations, speakers bureaus, manuscript writing, or educational events from Abbvie, Pfizer, and Teva; patents planned, issued, or pending (US 17/196,611); leadership or fiduciary role in other board, society, committee, or advocacy group, paid or unpaid with Abraces and IHS outside the submitted work. M.A.P. reports leadership or fiduciary role in other board, society, committee, or advocacy group, paid or unpaid as editor in chief of Annals of Clinical and Experimental Neurology outside the submitted work. P.P.-R. reports grants or contracts from ERANet Neuron, Instituto Salud Carlos III, and Novartis; consulting fees from AbbVie, Almirall, Dr. Reddy’s, Eli Lilly, Lundbeck, Organon, Pfizer, and Teva; payment or honoraria for lectures, presentations, speakers bureaus, manuscript writing, or educational events from AbbVie, Almirall, Dr. Reddy’s, Eli Lilly, Lundbeck, Novartis, Organon, Pfizer, and Teva; support for attending meetings and/or travel from Lundbeck, Organon, and Teva; and leadership or fiduciary role in other board, society, committee, or advocacy group, paid or unpaid with the Honorary Secretary International Headache Society outside the submitted work. J.I.S. reports other financial or non-financial support from the Yonsei Fellowship. J.S. reports consulting fees from ROMTech, Atheneum, Clearview healthcare partners, American College of Rheumatology, Yale, Hulio, Horizon Pharmaceuticals, DINO-RA, ANI/Exeltis, USA Inc., Frictionless Solutions, Schipher, Crealta/Horizon, Medisys, Fidia, PK Med, Two labs Inc., Adept Field Solutions, Clinical Care options, Putnam associates, Focus forward, Navigant consulting, Spherix, MedIQ, Jupiter Life Science, UBM LLC, Trio Health, Medscape, WebMD, and Practice Point communications, and the National Institutes of Health; payment or honoraria for lectures, presentations, speakers bureaus, manuscript writing, or educational events on the speaker’s bureau of Simply Speaking; support for attending meetings and/or travel from the speaker’s bureau of Simply Speaking; leadership or fiduciary role in other board, society, committee, or advocacy group, paid or unpaid as a Past steering committee member of the OMERACT, an international organization that develops measures for clinical trials and receives arm’s length funding from 12 pharmaceutical companies, as the Chair of the Veterans Affairs Rheumatology Field Advisory Committee, and as the editor and the Director of the UAB Cochrane Musculoskeletal Group Satellite Center on Network Meta-analysis; and stock or stock options in Atai life sciences, Kintara therapeutics, Intelligent Biosolutions, Acumen pharmaceutical, TPT Global Tech, Vaxart pharmaceuticals, Atyu biopharma, Adaptimmune Therapeutics, GeoVax Labs, Pieris Pharmaceuticals, Enzolytics Inc., Seres Therapeutics, Tonix Pharmaceuticals Holding Corp., Aebona Pharmaceuticals, and Charlotte’s Web Holdings, Inc., and previously owned stock options in Amarin, Viking, and Moderna pharmaceuticals outside the submitted work. M. Tanwar reports grants or contracts from the University of Alabama at Birmingham Health Service Foundation grant for TBI research outside the submitted work. M.Z. reports other financial support as an Alexion, AstraZeneca Rare Disease employee, outside the submitted work.

## STAR★Methods

### Key resources table

**Table:** 

REAGENT or RESOURCE	SOURCE	IDENTIFIER
**Deposited data**		

Data source of this paper	Global Burden of Diseases 2021. Available at https://vizhub.healthdata.org/gbd-results/	GBD 2021 is publicly available as of the date of publication.

**Software and algorithms**		

Python (version 3.12.8)	https://www.python.org/	Python software (version 3.12.8)
R (version 4.4.2)	https://www.r-project.org/	R (version 4.4.2)
Adobe Illustrator CC 2025	https://www.adobe.com/products/illustrator.html	Adobe company

### Experimental model and study participant details

The GBD 2021 provides comprehensive estimates of the burden of 371 diseases and injuries across 204 countries and territories from 1990 to 2021.^23^ It also offers estimates for seven GBD super-regions and 21 GBD regions, categorized by various health metrics such as prevalence and YLD. The data are disaggregated by sex, age, year, and location; however, stratification by gender, race, ethnicity, or ancestry was not available. This study adheres to the Guidelines for Accurate and Transparent Health Estimates Reporting (GATHER) statement (Table S3).^30^^,^^31^ Comprehensive descriptions of the methodology used for GBD estimation are available in prior publications.^23^ This research was conducted as part of the GBD Collaborator Network under the established GBD protocol.^11^

### Method details

#### Case definition and input data

GBD 2021 provides estimates for headaches categorized into specific subtypes, including migraine and TTH. These estimates are derived from survey data and literature sources related to migraine, TTH, and medication-overuse headaches (MOH).^23^ Detailed descriptions of all data sources, including PubMed search strategies, inclusion criteria for systematic reviews, and the number of sources used, are provided in the Figure S4.

Migraine is a disabling primary headache disorder typically characterized by recurrent, moderate to severe, unilateral, pulsatile headaches. Migraine is classified into two major types, with and without aura (transient neurological symptoms); however, GBD does not differentiate between them as most epidemiological studies report only on overall migraine. The reference diagnostic criteria for migraine are based on the ICHD-3.^23^ Estimates for migraine are derived from representative, population-based surveys and studies reporting on migraine.^16^ Systematic reviews for migraine input data were last conducted for GBD 2017, covering publications up to September 2017; studies based on medical claims were excluded due to their limited comparability with population-representative data. According to ICHD-3, a diagnosis of definite migraine requires fulfillment of five criteria, including attack duration of 4–72 h, specific headache characteristics (e.g., unilateral location, pulsating quality), and associated symptoms such as nausea or photophobia. Cases that meet all but one criterion—most commonly the duration requirement—are classified as probable migraine.^28^^,^^32^^,^^33^^,^^34^^,^^35^ Since GBD 2017, estimates have accounted for both definite and probable migraine by incorporating differences in case definitions across studies. Additionally, TTH is characterized by dull, non-pulsatile, diffuse, band-like (or vice-like) pain of mild to moderate intensity in the head or neck. The reference diagnostic criteria for TTH are also based on the ICHD-3. TTH estimates, like those for migraine, are derived from representative, population-based surveys and studies.^16^^,^^23^ A systematic review for TTH was also completed for GBD 2017 using identical inclusion criteria, with age- and sex-specific prevalence estimated or split using within-study ratios or pooled meta-analytic models. Migraine and TTH are treated as mutually exclusive in GBD analyses to prevent double counting. Each data point for migraine or TTH was labeled “definite” when all ICHD criteria were met and “probable” when exactly one was not; observations coded with earlier editions (ICHD-1 or ICHD-2) were converted to the ICHD-3 standard using version-specific cross-walk coefficients.^5^ According to ICHD-3, a diagnosis of definite TTH requires at least 10 episodes of headache lasting from 30 min to 7 days, accompanied by at least two of four features: bilateral location, pressing or tightening quality, mild to moderate intensity, and no aggravation by routine physical activity. Additionally, nausea or vomiting must be absent, and photophobia or phonophobia may be present but not both. As with migraine, a headache fulfilling all criteria is classified as definite TTH, and one that meets all but one criterion is classified as probable TTH.^23^ Prior to GBD 2017, estimates did not differentiate between probable and definite TTH; however, starting with GBD 2017, case definitions used across studies were incorporated to improve the accuracy and consistency of burden estimates.^23^ MOH was ascertained in two steps. First, respondents were screened for chronic headache (≥15 headache days per month for ≥3 months); second, follow-up questions captured regular over-use of acute headache medication as specified by ICHD-3.^5^^,^^23^ While MOH was included in the analysis to address the overlap between migraine and tension-type headaches, individual results for MOH are not presented separately.^16^^,^^23^ Input data for MOH were extracted from population-based prevalence studies. A meta-analysis was used to estimate the proportion of cases attributable to migraine and to TTH, allowing MOH to be classified into MOH-migraine and MOH-TTH.

#### Data processing and disease modeling

Both migraine and TTH were modeled in GBD 2021 with standard DisMod-MR settings. Excess mortality was set to zero, and it was assumed that there was no incidence or prevalence before the age of five. Each condition was categorized into probable, definite, and total, and separate DisMod-MR models were executed for each category. The results were then scaled to the total category to ensure consistency across estimates. For migraine, earlier data, particularly from before the adoption of the ICHD criteria, primarily reported on definite migraine.^23^ These data were adjusted to the total migraine category to improve modeling accuracy. Migraine and TTH were modeled separately using DisMod-MR 2.1, with estimates stratified into definite, probable, and total categories. Historical data predating the ICHD adoption were adjusted to align with the total envelope. Using MR-BRT regression, the proportion of symptomatic time was estimated at 9.3% for definite migraine and 6.6% for probable migraine. For TTH, unrealistic age patterns were observed in sex-specific regression models, necessitating the implementation of an age-based adjustment model for both sexes. Initially, sex-specific regression models were applied, but they produced unrealistic age patterns for females. To address this, a unified age-based adjustment was applied to both sexes, ensuring more accurate representation across age groups.^1^ MR-BRT regression estimated the symptomatic time proportion to be 2.9% for definite TTH and 2.1% for probable TTH.^16^^,^^23^ MOH was modeled separately in DisMod-MR (remission cap 0.4). Using meta-analytic data, 53.2% of the year was deemed symptomatic, and prevalence was split into MOH-migraine (73.2%) and MOH-TTH (26.8%). Prevalence was divided into MOH-migraine and MOH-TTH, with each subtype inheriting the DW of its respective parent condition (migraine or TTH). As MOH is defined as mutually exclusive from both migraine and TTH, no additional overlap adjustment was necessary; standard GBD comorbidity methods were applied.^1^

The GBD 2021 estimation process addresses data sparsity through hierarchical modeling and statistical borrowing across time, geography, and age. These adjustments help mitigate bias and enable robust estimation even in regions with limited input data.^23^ Furthermore, YLD is a quantitative measure of health loss caused by specific diseases or disabilities and plays a critical role in comparing disease burdens and prioritizing public health policies in GBD studies. YLD is calculated as the product of prevalence and DW, where prevalence is estimated using Bayesian modeling tools such as DisMod-MR, and DWs are derived from global surveys that measure disease severity on a scale from 0 (perfect health) to 1 (death).^31^ In calculating YLD, GBD stratifies data by sex, age, region, and year while independently evaluating the effects of comorbid conditions to avoid double counting. The calculation of YLD in GBD serves as an essential tool for global comparisons of disease burdens and for addressing regional health disparities.

#### Classification of geographic locations

GBD produced estimates for 204 countries and territories, organized into 21 regions and 7 super-regions. The seven super-regions are: Central Europe, Eastern Europe, and Central Asia, High-income, Latin America and the Caribbean, North Africa and the Middle East, South Asia, Southeast Asia, East Asia, and Oceania, and Sub-Saharan Africa. A full list of GBD regions is provided in Table S4. Similar to GBD 2019, GBD 2021 classifies areas as standard or non-standard. Standard GBD locations include all subnational areas from countries with populations exceeding 200 million and high-quality data. Subnational data for China, India, the US, and Brazil are considered standard locations, whereas Indonesia is not. China, India, the US, and Brazil are also included at the national level. All other countries with subnational estimations are classified as non-standard locations.

### Quantification and statistical analysis

#### Association between socio-economic indicators with the migraine and TTH

We used the SDI and the HAQ Index to examine the relationship between migraine, TTH, and socio-economic indicators. The SDI is a composite measure that reflects the social and economic conditions affecting health outcomes in each region. It is calculated as the geometric mean of three indices: the total fertility rate under age 25 years (TFU25), the average educational attainment of individuals aged 15 years and older (EDU15+), and the lagged distributive income per capita.^36^ The HAQ Index measures healthcare access and quality based on the mortality rates of conditions that should not result in death given effective medical care, providing insight into the performance of healthcare systems across regions.^16^ Both SDI and HAQ Index range from 0 to 1, where 0 indicates the lowest level of socio-demographic development and minimal healthcare access, respectively.

We examined the associations between the national SDI and HAQ Index and the age-standardized prevalence and YLD rates for migraine and TTH in 2021. Polynomial regression models were applied to capture the nonlinear relationships between these socioeconomic indicators and health outcomes.^37^

#### Estimate projections and decomposition analysis

We utilized the GBD 2021 forecasting framework to estimate the age-standardized YLD rates and all-age YLD numbers for migraine and TTH from 2022 to 2050. Non-fatal disease burden was forecasted using mixed-effects models, which were employed to directly model prevalence and incidence or to model the mortality-incidence ratio and mortality-prevalence ratio, converting forecasted mortality into incidence and prevalence. However, due to the lack of mortality data for migraine and TTH, the prevalence was directly modeled following the equation below:

$$logit\left(G_{l,a,s,y}\right)=\beta_{0}+\beta_{1}{SDI}_{l,y}+\pi_{0:l,a,s}+\epsilon_{l,a,s,y}$$

where $G_{l,a,s,y}$ represents the age-sex-location-year-specific prevalence, $\pi_{0:l,a,s}$ if the random intercept for age-sex-location, and $\epsilon_{l,a,s,y}$ is the residual term.^38^ From the modeled prevalence, YLDs were calculated by multiplying the estimated prevalence by the recent average DW. This approach assumes that DW remains constant over time, recognizing that future shifts in medical technologies and societal factors may introduce variability.^38^ The projected long-term trends through 2050 were generated using the internally optimized GBD forecasting framework, which applies scenario-based smoothing and calibration techniques to align with historical data patterns. Although formal back-testing procedures were not implemented, this approach is designed to produce reliable projections based on historical burden trajectories and demonstrated consistency across multiple disease domains.^39^

To examine the relative contributions of population growth, population aging, and changes in YLD rates for migraine and TTH between 2021 and 2050, the Das Gupta decomposition method was applied.^40^ To achieve this, we employed the decomposition methodology developed by Das Gupta, a widely recognized approach in demographic research for analyzing the impact of population structure and age distribution on health outcomes. This method enables a detailed quantitative evaluation of the contribution of each factor to variations in YLDs over time.^30^

The formula utilized in this analysis disaggregates the total YLDs into components attributable to population aging, overall population growth, and epidemiological changes. This approach provides critical insights into the underlying dynamics driving the burden of disease. The decomposition formula is expressed as follows:

$${YLDs}_{ay,py,ey}=\sum_{i=1}^{i=20}\left(a_{i,y}*p_{y}*e_{i,y}\right)$$

The total YLDs for migraine and TTH, denoted as ${YLDs}_{ay,py,ey}$*,* are calculated as a function of age distribution, total population size, and age-specific YLD rates. In this formulation, i represents discrete age groups, and y denotes the observation year ranging from 2021 to 2050. The term $a_{i,y}$ indicates the proportion of the population, $p_{y}$ refers to the total population size, and $e_{i,y}$ corresponds to the YLD rate for the specific condition (migraine or TTH) within. This formulation enables the decomposition of total YLDs into components attributable to demographic structure—specifically population growth and aging—and epidemiological changes. By algebraically disaggregating these standardized effects, the analysis quantifies the relative contribution of each factor to the overall change in disease burden.^41^

#### Data presentation

The age-standardized rates for prevalence and YLD of headache disorders were calculated using the GBD world population age standard.^42^ Unless stated as an age-specific rate, all rates are presented in age-standardized rates. To evaluate temporal changes (e.g., 1990 to 2021), we determined the percentage change, with the difference between the end and start rates divided by the start rate. Final point estimates are reported with 95% UIs. The UIs represent the range within which the true value is expected to fall. They were calculated as the 2.5^th^ and 97.5^th^ percentile of the distribution of 500 draws at each step in the migraine and TTH estimation process. These 500 draws were used to propagate uncertainty arising from multiple sources, including variability in input data, model parameters, and DWs. Uncertainty was systematically incorporated and propagated throughout the modeling process in accordance with the standard GBD estimation framework.^23^^,^^43^ All analyses were conducted using Python software (version 3.12.8; Python Software Foundation, Wilmington, DE, USA) and R (version 4.4.2; R Foundation, Vienna, Austria). The full affiliations of the GBD 2021 Headache Collaborators are provided in Data S1.

## Supplemental information

Document S1. Figures S1–S4 and Tables S3 and S4

Table S1. Age-standardized prevalence, YLD, and incidence rates of migraine in 2021 and percentage change (1990–2021) by country and globally (95% UI), related to Figure 1 Abbreviation: UI, uncertainty interval; YLD, years lived with disability.

Table S2. Age-standardized prevalence, YLD, and incidence rates of TTH in 2021 and percentage change (1990–2021) by country and globally (95% UI), related to Figure 1 Abbreviation: TTH, tension-type headaches; UI, uncertainty interval; YLDs, years lived with disability.

Data S1. Affiliations of individual authors, related to STAR Methods

Document S2. Article plus supplemental information
