# Supplementary material for: Global, regional, and national burden of headache disorders, 1990–2021, with forecasts to 2050: A Global Burden of Disease study 2021
Source: Cell Rep Med. 2025 Sep 18;6(10):102348. doi: 10.1016/j.xcrm.2025.102348 (PMC12629824; doi:10.1016/j.xcrm.2025.102348)
Supplement: Document S1. Figures S1–S4 and Tables S3 and S4 [file mmc1.pdf]

Cell Reports Medicine, Volume *6*

Supplemental information

Global, regional, and national burden of headache disorders, 1990–2021, with forecasts to 2050: A Global Burden of Disease study 2021

GBD 2021 Headache Collaborators, Tissa Wijeratne, Jiyeon Oh, Soeun Kim, Yesol Yim, Min Seo Kim, Jae Il Shin, Yun-Seo Oh, Raon Jung, Yun Seo Kim, Lee Smith, Hasan Aalruz, Rami Abd-Rabu, Deldar Morad Abdulah, Richard Gyan Aboagye, Meysam Abolmaali, Dariush Abtahi, Ahmed Abualhasan, Rufus Adesoji Adedoyin, Qorinah Estiningtyas Sakilah Adnani, Fatemeh Afrashteh, Navidha Aggarwal, Danish Ahmad, Ali Ahmadi, Negar Sadat Ahmadi, Amir Mahmoud Ahmadzade, Syed Anees Ahmed, Salah Al Awaidy, Sawsan Alabbad, Muaaz M. Alajlani, Yazan Al-Ajlouni, Mohammed Usman Ali, Syed Shujait Ali, Waad Ali, Joseph Uy Almazan, Najim Z. Alshahrani, Awais Altaf, Mohammad Al-Wardat, Karem H. Alzoubi, Sohrab Amiri, Hubert Amu, Ganiyu Adeniyi Amusa, David B. Anderson, Saleha Anwar, Demelash Areda, Mohammad Asghari-Jafarabadi, Sait Ashina, Javed Ashraf, Tahira Ashraf, Ali Azargoonjahromi, Yogesh Bahurupi, Atif Amin Baig, Soham Bandyopadhyay, Mainak Bardhan, Hiba Jawdat Barqawi, Azadeh Bashiri, Mohammad-Mahdi Bastan, Maryam Bemanalizadeh, Isabela M. Bensenor, Alemshet Yirga Yirga Berhie, Akshaya Srikanth Bhagavathula, Sonu Bhaskar, Vivek Bhat, Gurjit Kaur Bhatti, Jasvinder Singh Bhatti, Cem Bilgin, Atanu Biswas, Bruno Bizzozero-Peroni, Yasser Bustanji, Luis Alberto Cámera, Edoardo Caronna, Andre F. Carvalho, Sandip Chakraborty, Patrick R. Ching, Nikos Christodoulou, Dinh-Toi Chu, Hongyuan Chu, Natalia Cruz-Martins, Omid Dadras, Xiaochen Dai, Emanuele D'Amico, Amira Hamed Darwish, Sindhura Deekonda, Vinoth Gnana Chellaiyan Devanbu, Samath Dhamminda Dharmaratne, Adriana Dima, Temesgien Ergetie Dinkayehu, Huyen Do, Paul Narh Doku, Ojas Prakashbhai Doshi, Abdel Rahman E'mar, Negin Eissazade, Chadi Eltaha, Ayesha Fahim, Jawad Fares, Mohsen Farjoud Kouhanjani, Andre Faro, Patrick Fazeli, Seyed-Mohammad Fereshtehnejad, Pietro Ferrara, Nuno Ferreira, Florian Fischer, Arianna Fornari, Márió Gajdács, Miglas Welay Gebregergis, Delaram J. Ghadimi, Amir Ghaffari Jolfayi, Elena V. Gnedovskaya, Mahaveer Golechha, Enrique Gomez Figueroa, Mohammad Hashem Hashempur, Md Saquib Hasnain, Amr Hassan, Nageeb Hassan, Mahgol Sadat Hassan Zadeh Tabatabaei, Mohamed I. Hegazy, Golnaz Heidari, Bartosz Helfer, Md Mahbub Hossain, Mowafa Househ, Chengxi Hu, Ivo Iavicoli, Olayinka Stephen Ilesanmi, Irena M. Ilic, Muhana Fawwazy Ilyas, Salim Ilyasu, Nahlah Elkudssiah Ismail, Ali Jafari-Khounigh, Haitham Jahrami, Manthan Dilipkumar Janodia, Ruwan Duminda Jayasinghe, Bijay Mukesh Jeswani, Jost B. Jonas, Nitin Joseph, Rizwan Kalani, Moien A.B. Khan, Sorour Khateri, Mahalaqua Nazli Khatib, Hamid Reza Khayat Kashani, Feriha Fatima Khidri, Moein Khormali, Sepehr Khosravi, Yun Jin Kim, Farzad Kompani, Karel Kostev, Kewal Krishan, Bindu Krishnan, Barthelemy Kuate Defo, Mohammed

1. Supplementary Figures

Figure S1. Association between age-standardized YLD rate of migraine and TTH and HAQ index by GBD super regions. Related to Figure 3.

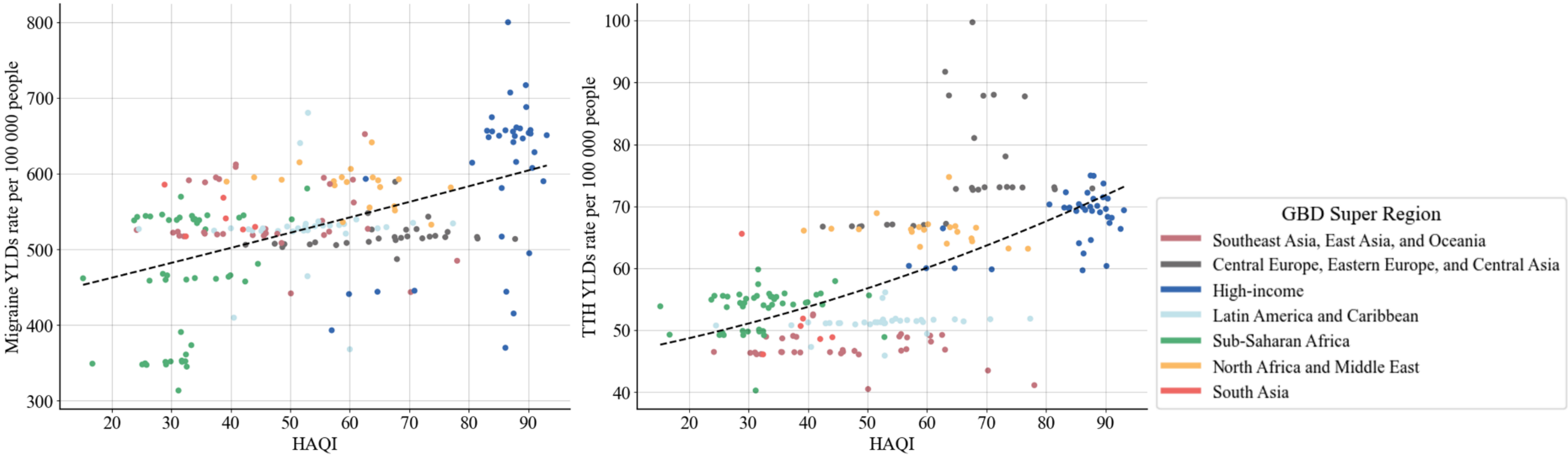

Abbreviation: GBD, Global Burden of Diseases, Injuries, and Risk Factors; HAQ, Healthcare Access Quality; TTH, tension-type headaches; YLD, years lived with disability.

Figure S2. Association between age-standardized prevalence rate of migraine and TTH with SDI and HAQI by GBD super regions. Related to Figure 3.

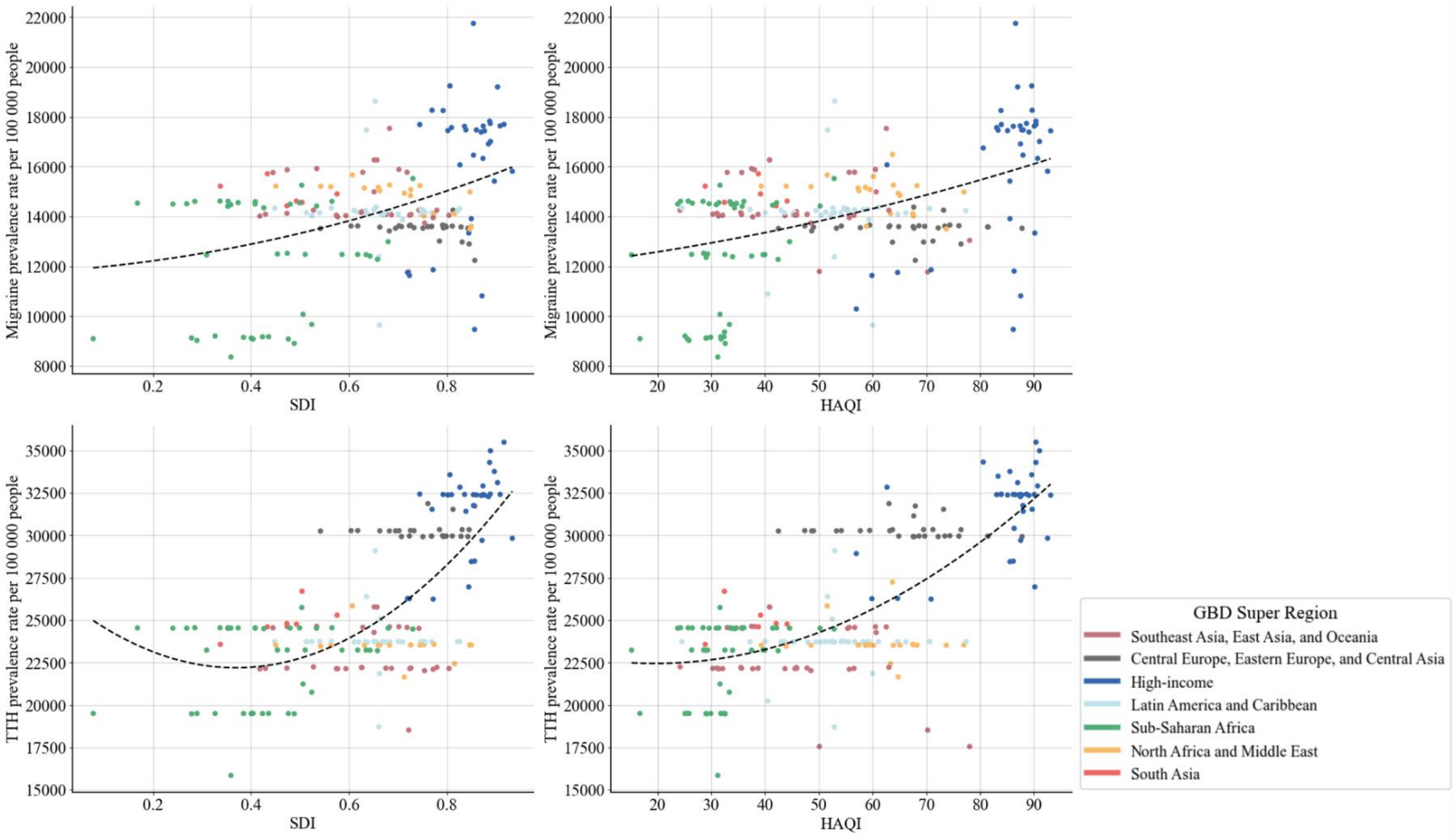

Abbreviation: GBD, Global Burden of Diseases, Injuries, and Risk Factors; HAQI, Healthcare Access Quality Index; SDI, socio-demographic index; TTH, tension-type headaches; YLD, years lived with disability.

Figure S3. Age-standardized YLD and prevalence rates for migraine and TTH by SDI levels. Related to Figure 3.

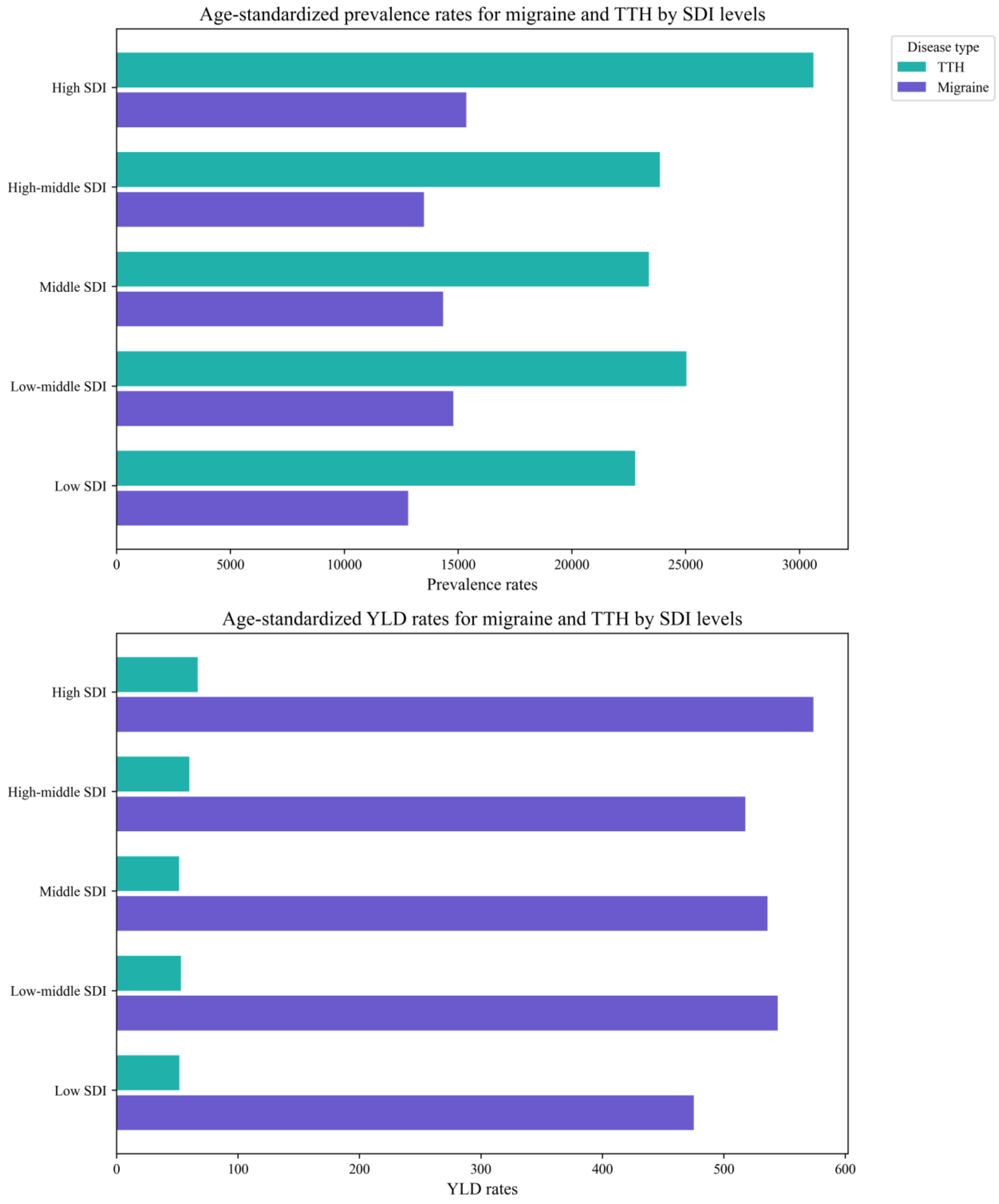

Abbreviation: SDI, socio-demographic index; TTH, tension-type headaches; YLD, years lived with disability.

Figure S4. Flowchart of headache. Related to STAR Methods.

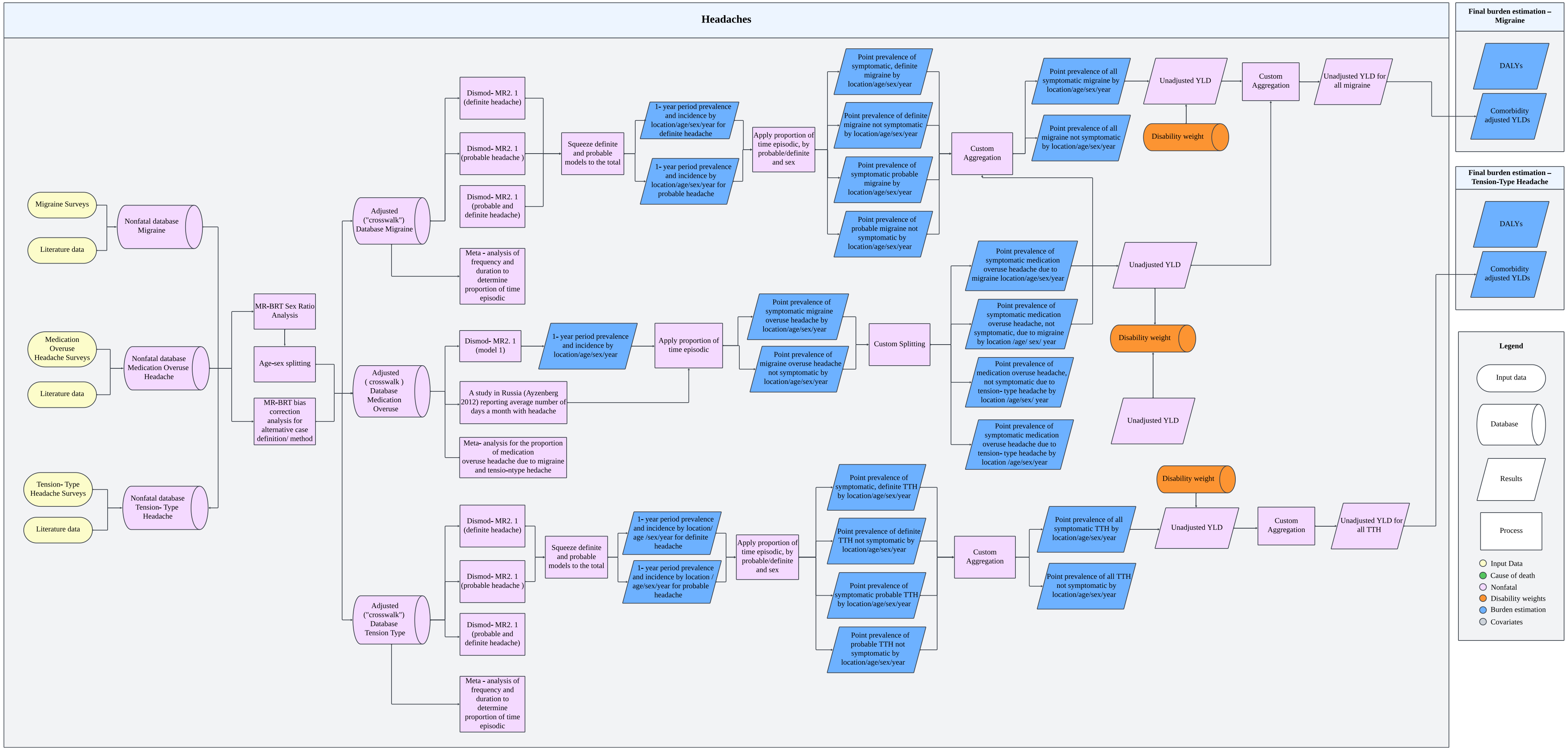

3. Supplementary Tables

Table S3. Guidelines for GATHER. Related to STAR Methods.

This study adheres to the Guidelines for Accurate and Transparent Health Estimates Reporting (GATHER) recommendations, as demonstrated by the inclusion of the GATHER checklist.

Item #	Checklist item	Reported on page #
**Objectives and funding**		
**1**	Define the indicator(s), populations (including age, sex, and geographic entiti es), and time period(s) for which estimates were made.	Introduction and Methods
**2**	List the funding sources for the work.	Introduction and Methods
**Data Inputs**		
For all data inputs from multiple sources that are synthesized as part of the study:		
**3**	Describe how the data were identified and how the data were accessed.	Methods
**4**	Specify the inclusion and exclusion criteria. Identify all ad-hoc exclusions.	Methods
**5**	Provide information on all included data sources and their main characteristi cs. For each data source used, report reference information or contact name/ institution, population represented, data collection method, year(s) of data col lection, sex and age range, diagnostic criteria or measurement method, and s ample size, as relevant.	Methods
**6**	Identify and describe any categories of input data that have potentially impo rtant biases (e.g., based on characteristics listed in item 5).	Methods
For data inputs that contribute to the analysis but were not synthesized as part of the study:		
**7**	Describe and give sources for any other data inputs.	Methods
For all data inputs:		
**8**	Provide all data inputs in a file format from which data can be efficiently extracted (e.g., a spreadsheet rather than a PDF), including all relevant meta -data listed in item 5. For any data inputs that cannot be shared because of ethical or legal reasons, such as third-party ownership, provide a contact n ame or the name of the institution that retains the right to the data.	Methods
**Data analysis**		
**9**	Provide a conceptual overview of the data analysis method. A diagram may be helpful.	Methods
**10**	Provide a detailed description of all steps of the analysis, including mathem atical formulae. This description should cover, as relevant, data cleaning, dat a pre-processing, data adjustments and weighting of data sources, and mathe matical or statistical model(s).	Methods
**11**	Describe how candidate models were evaluated and how the final model(s) were selected.	Methods

**12**	Provide the results of an evaluation of model performance, if done, as well as the results of any relevant sensitivity analysis.	Methods
**13**	Describe methods for calculating uncertainty of the estimates. State which so urces of uncertainty were, and were not, accounted for in the uncertainty an alysis.	Methods
**14**	State how analytic or statistical source code used to generate estimates can be accessed.	Methods
**Results and Discussion**		
**15**	Provide published estimates in a file format from which data can be efficie ntly extracted.	Results and Discussion
**16**	Report a quantitative measure of the uncertainty of the estimates (e.g. uncert ainty intervals).	Results and Discussion
**17**	Interpret results in light of existing evidence. If updating a previous set of estimates, describe the reasons for changes in estimates.	Results and Discussion
**18**	Discuss limitations of the estimates. Include a discussion of any modelling a ssumptions or data limitations that affect interpretation of the estimates.	Discussion

Table S4. Classification of geographic locations. Related to STAR Methods.

GBD super regions	GBD regions	Country
Southeast Asia, East Asia, and Oceania	East Asia	China, Democratic People's Republic of Korea, Taiwan (Province of China)
	Oceania	American Samoa, Cook Islands, Fiji, Guam, Kiribati, Marshall Islands, Micronesia, Nauru, Niue, Northern Mariana Islands, Palau, Papua New Guinea, Samoa, Solomon Islands, Tokelau, Tonga, Tuvalu, Vanuatu
	Southeast Asia	Cambodia, Indonesia, Lao People's Democratic Republic, Malaysia, Maldives, Mauritius, Myanmar, Philippines, Seychelles, Sri Lanka, Thailand, Timor-Leste, Viet Nam
Central Europe, Eastern Europe, and Central Asia	Central Asia	Armenia, Azerbaijan, Georgia, Kazakhstan, Kyrgyzstan, Mongolia, Tajikistan, Turkmenistan, Uzbekistan
	Central Europe	Albania, Bosnia and Herzegovina, Bulgaria, Croatia, Czechia, Hungary, Montenegro, North Macedonia, Poland, Romania, Serbia, Slovakia, Slovenia
	Eastern Europe	Belarus, Estonia, Latvia, Lithuania, Republic of Moldova, Russian Federation, Ukraine
High-income	Australasia	Australia, New Zealand
	High-income Asia Pacific	Brunei Darussalam, Japan, Republic of Korea, Singapore
	High-income North America	Canada, Greenland, United States of America
	Southern Latin America	Argentina, Chile, Uruguay
	Western Europe	Andorra, Austria, Belgium, Cyprus, Denmark, Finland, France, Germany, Greece, Iceland, Ireland, Israel, Italy, Luxembourg, Malta,

		Monaco, Netherlands, Norway, Portugal, San Marino, Spain, Sweden, Switzerland, United Kingdom
Latin America and Caribbean	Andean Latin America	Bolivia, Ecuador, Peru
	Caribbean	Antigua and Barbuda, Bahamas, Barbados, Belize, Bermuda, Cuba, Dominica, Dominican Republic, Grenada, Guyana, Haiti, Jamaica, Puerto Rico, Saint Kitts and Nevis, Saint Lucia, Saint Vincent and the Grenadines, Suriname, Trinidad and Tobago, United States Virgin Islands
	Central Latin America	Colombia, Costa Rica, El Salvador, Guatemala, Honduras, Mexico, Nicaragua, Panama, Venezuela (Bolivarian Republic of)
	Tropical Latin America	Brazil, Paraguay
North Africa and Middle East	North Africa and Middle East	Afghanistan, Algeria, Bahrain, Egypt, Iran, Iraq, Jordan, Kuwait, Lebanon, Libya, Morocco, Oman, Palestine, Qatar, Saudi Arabia, Sudan, Syrian Arab Republic, Tunisia, Türkiye, United Arab Emirates, Yemen
South Asia	South Asia	Bangladesh, Bhutan, India, Nepal, Pakistan
Sub-Saharan Africa	Central sub-Saharan Africa	Angola, Central African Republic, Congo, Democratic Republic of the Congo, Equatorial Guinea, Gabon
	Eastern sub-Saharan Africa	Burundi, Comoros, Djibouti, Eritrea, Ethiopia, Kenya, Madagascar, Malawi, Mozambique, Rwanda, Somalia, South Sudan, Uganda, United Republic of Tanzania, Zambia
	Southern sub-Saharan Africa	Botswana, Eswatini, Lesotho, Namibia, South Africa, Zimbabwe

	Western sub-Saharan Africa	Benin, Burkina Faso, Cabo Verde, Cameroon, Chad, Cote d'Ivoire, Gambia, Ghana, Guinea, Guinea-Bissau, Liberia, Mali, Mauritania, Niger, Nigeria, Sao Tome and Principe, Senegal, Sierra Leone, Togo
